# Single-Cell and Machine Learning Analyses Identify a PFKFB3-Centered Regulatory Network and Potential Salidroside Interaction in Coronary Heart Disease

**DOI:** 10.3390/ijms27167413

**Published:** 2026-08-19

**Authors:** Haobo Yang, Yonghui Zhang, Yunfeng Yu, Yanan Bai, Jiale Zhu, Ouying Chen, Liping Wang, Weixiong Jian

**Affiliations:** 1 School of Traditional Chinese Medicine, Hunan University of Chinese Medicine, Changsha 410208, China; daxiong990420@163.com (H.Y.); zzyy040699@163.com (Y.Z.); yuyunfeng1510@163.com (Y.Y.); daxiong1020@163.com (Y.B.); 004687@hnucm.edu.cn (J.Z.); 2 Hunan Provincial Key Laboratory of TCM Diagnostics, Institute of National Key Discipline in TCM Diagnostics, Changsha 410208, China; 3 School of Nursing, Hunan University of Traditional Chinese Medicine, Changsha 410208, China; 4 School of Traditional Chinese Medicine, Hunan University of Medicine, Huaihua 418000, China; wangliping@humu.edu.cn

**Keywords:** coronary heart disease, single-cell transcriptomics, machine learning, PFKFB3, salidroside, molecular dynamics simulation, immunometabolism, diagnostic model

## Abstract

Coronary heart disease (CHD) is a leading cause of morbidity and mortality, driven by metabolic remodeling, vascular inflammation, and perivascular adipose tissue (PVAT) dysfunction. We integrated bulk transcriptomic datasets to develop a machine learning-based diagnostic model, evaluated 113 algorithms, and identified a seven-gene signature (*PYGL*, *PTGS2*, *PFKFB3*, *MMP9*, *CYP1B1*, *CXCR1*, *ABCB1*) with robust predictive performance. Single-cell RNA sequencing (scRNA-seq) of coronary PVAT revealed substantial cellular heterogeneity and prioritized PFKFB3 as a hub linking glycolytic activity to nuclear factor kappa B (NF-κB) regulon activity. Macrophage-centered communication via secreted phosphoprotein 1 (SPP1), migration inhibitory factor (MIF), and other pathways was enhanced in disease conditions. Virtual knockout of PFKFB3 induced transcriptional changes enriched in immune activation, phagocytosis, and oxidative stress, while molecular dynamics (MD) simulations suggested that salidroside can adopt a stable binding pose within the PFKFB3 pocket, providing structural plausibility for their interaction. Together, these analyses provide a multi-layered framework connecting glycolytic remodeling, inflammatory transcriptional activity, and intercellular signaling in CHD. The findings support PFKFB3 as a potential biomarker and mechanistic hub and suggest that salidroside may modulate its activity. This study offers an integrative computational foundation for future experimental validation and mechanistic exploration of PVAT dysfunction in CHD.

## 1. Introduction

Coronary heart disease (CHD) remains a predominant cause of global morbidity and mortality, driven not only by traditional risk factors such as dyslipidemia and hypertension but increasingly by perturbations in cellular metabolism and inflammation [1]. Recent advances have underscored the role of metabolic reprogramming—particularly enhanced glycolytic flux—as a hallmark of vascular and immune cell dysfunction in cardiovascular pathology [2,3]. Central to this metabolic shift is 6-phosphofructo-2-kinase/fructose-2,6-bisphosphatase 3 (PFKFB3), a bifunctional enzyme that catalyzes the synthesis of fructose-2,6-bisphosphate, a potent allosteric activator of phosphofructokinase-1 and a key driver of glycolytic throughput [4]. Elevated PFKFB3 expression and activity have been linked to endothelial phenotypic changes, inflammatory activation, and vascular remodeling in cardiopulmonary disease models, suggesting that aberrant glycolytic metabolism may sustain pathological processes in CHD and related ischemic conditions [5]. The importance of PFKFB3 in modulating the metabolic–inflammatory axis is increasingly appreciated, with evidence that its dysregulation contributes to endothelial dysfunction, macrophage activation, and adverse remodeling, positioning PFKFB3 as both a biomarker and a potential target for further investigation in cardiometabolic disease states [6].

Despite advances in understanding the metabolic underpinnings of cardiovascular disease, effective strategies to modulate pathological glycolysis in CHD remain under development [4,5]. In parallel, there has been growing interest in natural compounds with pleiotropic bioactivities that may intersect with metabolic and inflammatory pathways relevant to CHD [7]. Salidroside, a phenolic glycoside derived from Rhodiola species, has gained attention for its adaptogenic, anti-inflammatory, antioxidative, and tissue-protective properties, particularly in ischemic and metabolic disorders. Pharmacological studies indicate that salidroside can enhance cell survival, promote angiogenesis, and ameliorate oxidative and inflammatory injury in multiple organ systems, including the heart, brain, and skeletal muscle, through modulation of signaling cascades involved in cellular survival and stress responses [5]. Pharmacology analyses specifically identify salidroside’s potential efficacy in coronary artery disease, where it may influence angiogenic mediators and vascular function, although its direct impact on glycolytic regulators such as PFKFB3 has not been systematically characterized in CHD contexts [4]. Given the convergence of glycolytic reprogramming and vascular inflammation in CHD, together with salidroside’s reported cardiovascular protective effects [8,9], we hypothesized that salidroside may interact with PFKFB3 to modulate glycolytic regulation.

To test this hypothesis, we employed a stepwise analytical framework: (1) identification of CHD-associated DEGs from bulk transcriptomic data, (2) construction and validation of a machine learning-based diagnostic model, (3) single-cell resolution mapping of diagnostic genes in PVAT, (4) prioritization of a key regulatory hub gene coupled with metabolic pathway and transcription factor analyses, (5) characterization of intercellular communication remodeling, (6) in silico perturbation and functional enrichment of the hub gene network, (7) molecular dynamics simulation to assess the structural plausibility of a candidate interaction,(8) integrative mechanistic modeling.

## 2. Results

### 2.1. Identification of Differentially Expressed Genes Associated with CHD

**Figure 1:**
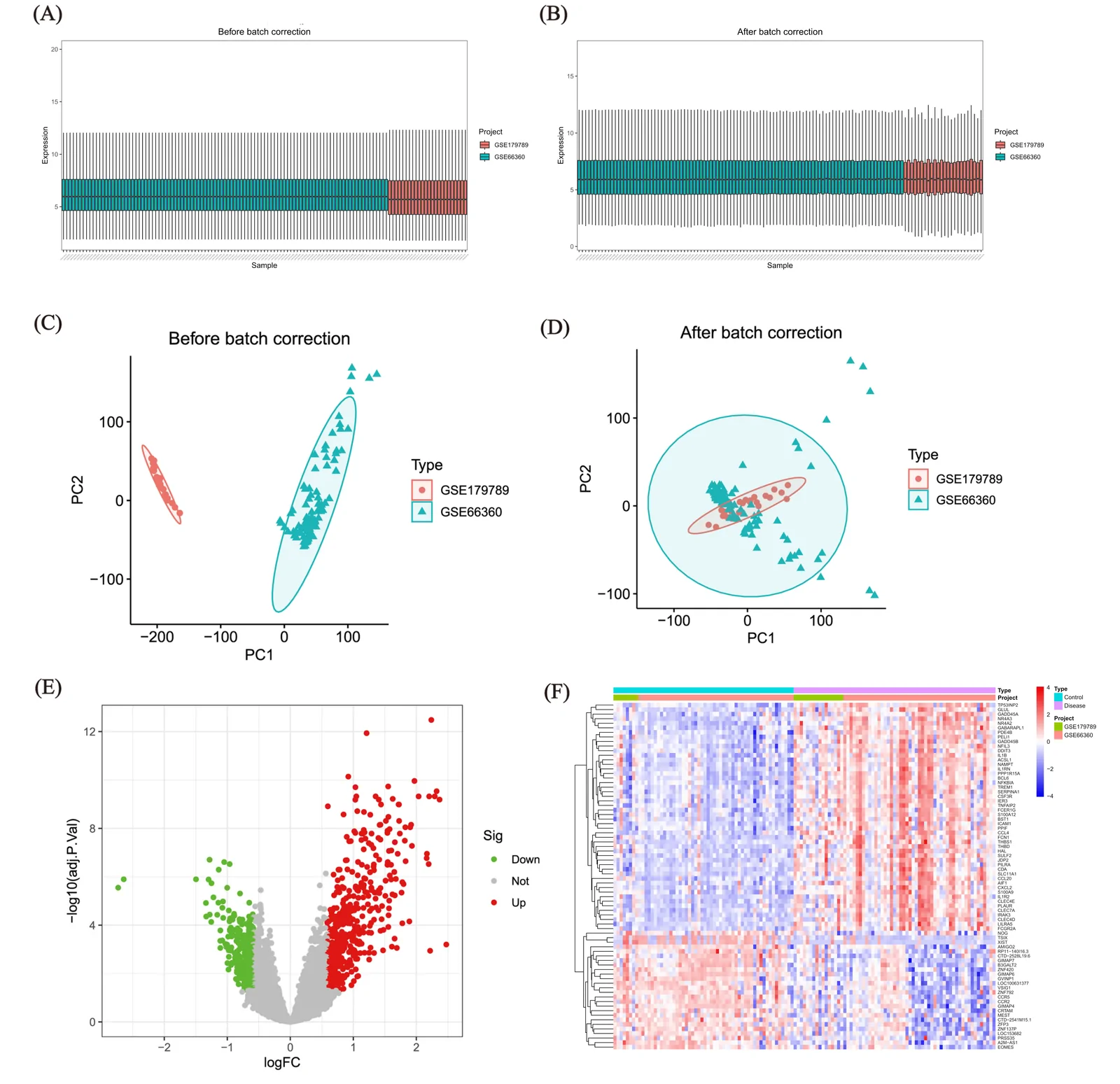
Data preprocessing and differential expression analysis. (**A**,**B**) Box plot comparison of gene expression levels before and after batch calibration. (**C**,**D**) Sample distribution maps based on PCA before and after batch calibration. (**E**) Volcano map of DEGs. (**F**) Hierarchical clustering heatmap.

To identify transcriptomic alterations associated with coronary heart disease (CHD), the GSE66360 and GSE179789 datasets were merged to construct the training cohort and subsequently subjected to batch effect correction [10,11]. Before correction, the overall expression distributions of the two datasets were not fully comparable, and PCA showed clear separation between samples from different datasets, indicating a substantial batch effect (Figure 1A,C). After ComBat correction, the expression distributions became more consistent across samples (Figure 1B), and the PCA plot showed markedly improved overlap between the two datasets (Figure 1D), suggesting that batch effects had been effectively reduced and that the merged dataset was suitable for downstream integrative analysis. Based on the batch-corrected training cohort, differential expression analysis was performed between the CHD and control groups using the thresholds of false discovery rate (FDR) < 0.05 and |log2 fold change (log2FC)| > 1. As shown in the volcano plot, a substantial number of genes were significantly dysregulated in CHD, including both upregulated and downregulated transcripts (Figure 1E). In total, 769 differentially expressed genes (DEGs) were identified, indicating widespread transcriptional alterations associated with CHD. To further evaluate the overall expression pattern of these DEGs, hierarchical clustering analysis was performed and visualized as a heatmap. The identified DEGs exhibited distinct expression profiles between CHD and control samples, with clear clustering trends observed between the two groups (Figure 1F ). Collectively, these results demonstrated that CHD is associated with significant transcriptomic remodeling and provided the basis for subsequent machine learning-based feature selection and downstream mechanistic analyses.

### 2.2. Construction of a Machine Learning Model and Identification of a Seven-Gene Diagnostic Signature

**Figure 2:**
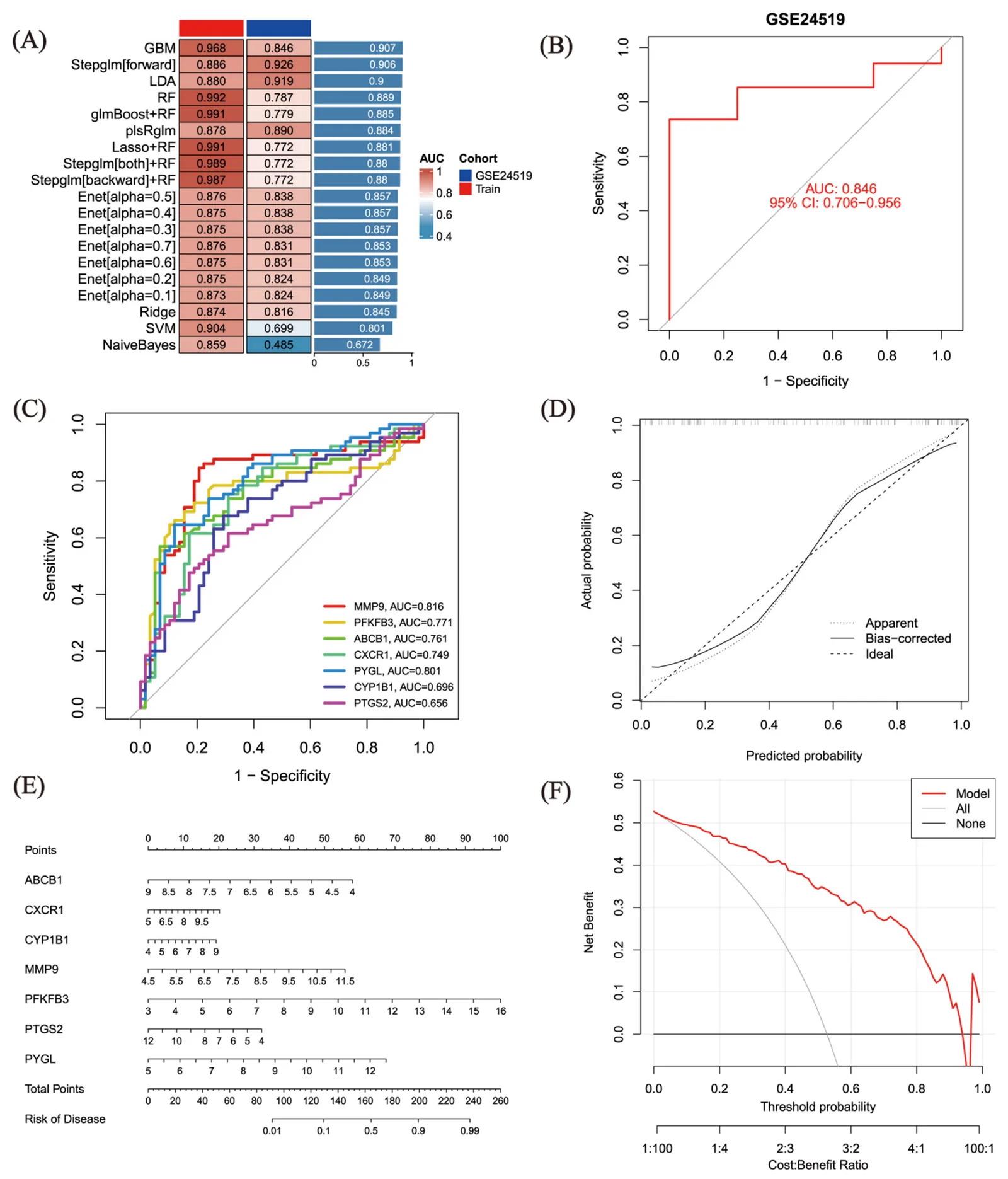
Construction and multi-dimensional evaluation of a machine learning diagnostic model based on core targets. (**A**) Performance Comparison of 113 Machine Learning Algorithms in the Training Set. (**B**) Receiver Operating Characteristic (ROC) curves of GBM model in training set and independent validation set. (**C**) Bar chart of AUC values of each core target as independent biomarkers in the validation set. (**D**) Nomogram of the diagnostic model. (**E**) Calibration curve of the diagnostic model. (**F**) DCA of the Model.

Having identified transcriptomic alterations associated with CHD, we next sought to determine whether these differentially expressed genes could be leveraged to construct a robust diagnostic classifier. A total of 113 machine learning algorithms, comprising combinations of feature selection methods (LASSO, glmBoost, Random Forest, and Stepwise GLM) and classifiers (Gradient Boosting Machine, Random Forest, Support Vector Machine, Linear Discriminant Analysis, XGBoost, Naive Bayes, Ridge regression, LASSO, Elastic Net with α = 0.1–0.9, Stepwise GLM [forward, backward, and both directions], glmBoost, and partial least squares regression for GLM), were trained and compared in the training cohort using five repeats of ten-fold cross-validation. The complete list of all 113 algorithm combinations is provided in Supplementary Table S2, and their full performance metrics are provided in Supplementary Table S3. Among the evaluated models, the Gradient Boosting Machine (GBM) achieved the highest average AUC (0.910) across the training cohort (AUC = 0.975) and the independent validation cohort (AUC = 0.846, 95% CI: 0.709–0.956). The top five performing algorithms were: GBM (average AUC = 0.910), RF + GBM (0.910), Stepwise GLM [forward] (0.906), RF + Stepwise GLM [forward] (0.906), and Linear Discriminant Analysis (0.900) (Figure 2A).

Based on the optimal model, a seven-gene diagnostic signature consisting of *PYGL, PTGS2, PFKFB3, MMP9, CYP1B1, CXCR1*, and *ABCB1* was established. In the independent validation cohort, the GBM model achieved an AUC of 0.846 (95% CI: 0.709–0.956), indicating good discriminatory ability for distinguishing CHD samples from controls (Figure 2B). To further evaluate the contribution of each component gene, ROC analysis was also performed for the seven genes individually in the validation cohort (Figure 2C). To assess model robustness and the potential risk of overfitting, the training and validation AUCs of all algorithms were compared. The Random Forest model exhibited the largest Train–Validation AUC gap (0.991 vs. 0.787, Δ = 0.204), consistent with its known tendency to overfit on small feature sets. In contrast, GBM demonstrated a substantially smaller gap (0.975 vs. 0.846, Δ = 0.129), indicating better generalizability. The top five algorithms all converged on the same seven-gene signature with validation AUCs > 0.84, suggesting that the diagnostic performance is driven by the selected gene features rather than by any single classifier, and that the risk of model-specific overfitting is low. The complete list of 203 differentially expressed genes (|log_2_FC| > 1, FDR < 0.05) used as candidate features for machine learning is provided in Supplementary Table S1.

To facilitate individualized risk estimation, a nomogram based on the diagnostic signature was constructed (Figure 2D). The corresponding calibration curve showed good agreement between the predicted and observed probabilities (Figure 2E), suggesting acceptable calibration performance of the model. In addition, decision curve analysis (DCA) indicated that the model provided a positive net clinical benefit across a range of threshold probabilities compared with the “treat-all” or “treat-none” strategies (Figure 2F). Collectively, these results suggest that the seven-gene signature has potential diagnostic value for CHD and provides a practical framework for subsequent biological interpretation.

### 2.3. Single-Cell Transcriptomic Profiling Reveals the Cellular Landscape of PVAT in CHD

**Figure 3:**
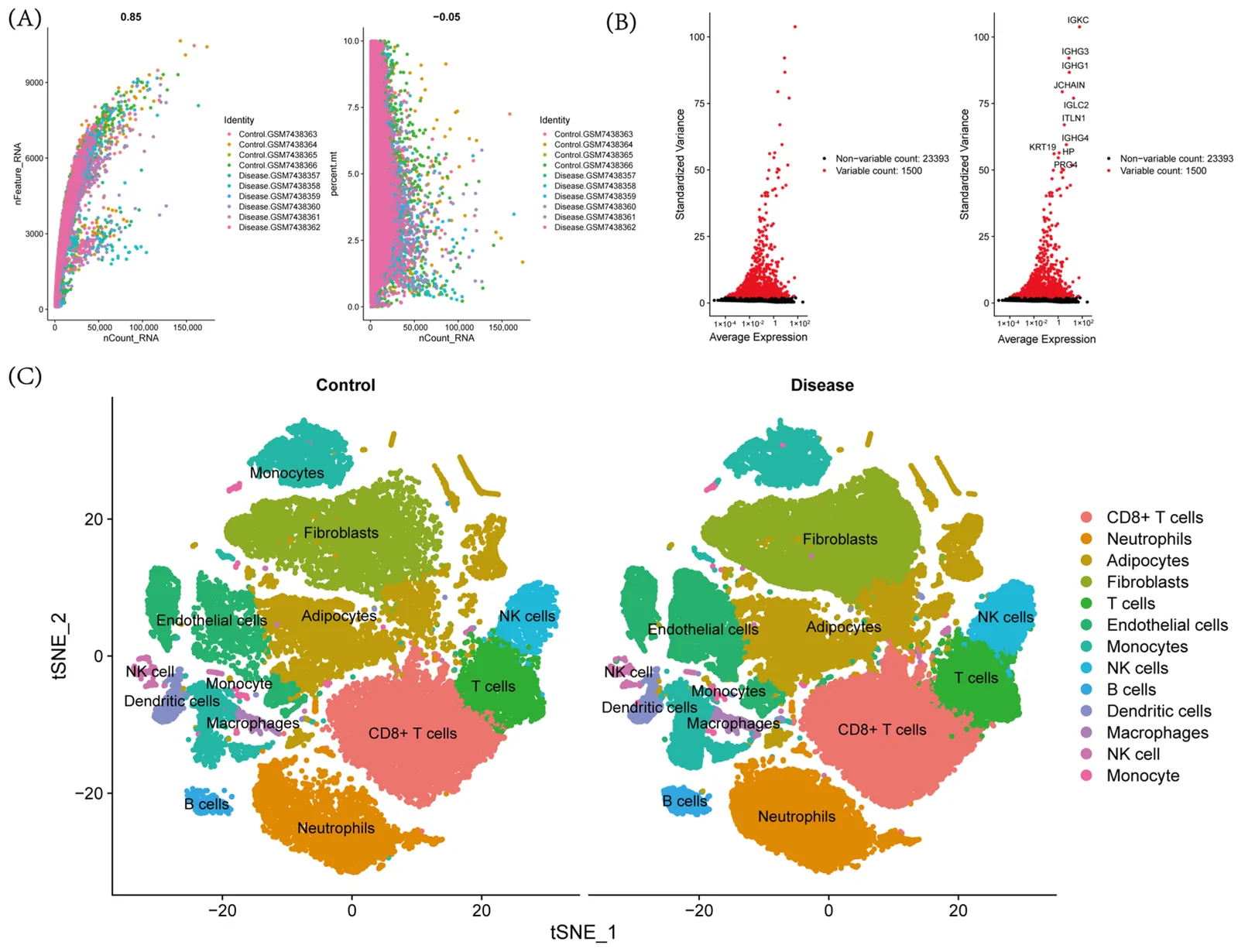
Single-cell transcriptomic profiling of PVAT in CHD. (**A**) Quality control plots showing the relationships among detected genes, transcript counts, and mitochondrial gene proportions in the single-cell dataset. (**B**) Highly variable gene selection plots used for downstream dimensionality reduction and clustering. (**C**) t-SNE plots showing the distribution of major cell populations in PVAT from the control and disease groups. Cell clusters were annotated based on canonical marker genes.

Although the seven-gene diagnostic signature showed promising performance in bulk transcriptomic data, bulk profiling cannot resolve the cellular context in which these genes operate. To address this limitation, we turned to single-cell transcriptomics. To characterize the cellular heterogeneity of PVAT in CHD, single-cell RNA sequencing data from GSE233870 were analyzed after quality control, normalization, and clustering. Quality control assessment showed acceptable relationships among the number of detected genes, transcript counts, and mitochondrial gene proportions across cells, indicating that the dataset was suitable for downstream single-cell analysis (Figure 3A). In addition, highly variable gene analysis identified genes with marked expression variability for subsequent dimensionality reduction and clustering (Figure 3B). Following data preprocessing and clustering, the cells were projected into a low-dimensional space using t-SNE. The t-SNE plots revealed a well-resolved cellular landscape in PVAT and showed broadly comparable major cell populations in both the control and disease groups (Figure 3C). Based on canonical marker genes, multiple major cell populations were identified, including CD8+ T cells, neutrophils, adipocytes, fibroblasts, T cells, endothelial cells, monocytes, NK cells, B cells, dendritic cells, macrophages, and monocyte-like cell populations. Among these, CD8+ T cells, neutrophils, fibroblasts, adipocytes, and T cells constituted the major cellular components, whereas immune cell subsets such as monocytes, macrophages, dendritic cells, NK cells, and B cells were also clearly detected.

Overall, these results demonstrate substantial cellular heterogeneity in PVAT and provide a single-cell framework for subsequent analysis of the cell-type-specific expression patterns of the diagnostic genes in CHD.

### 2.4. Prioritization of PFKFB3 as a Key Regulatory Hub Gene

**Figure 4:**
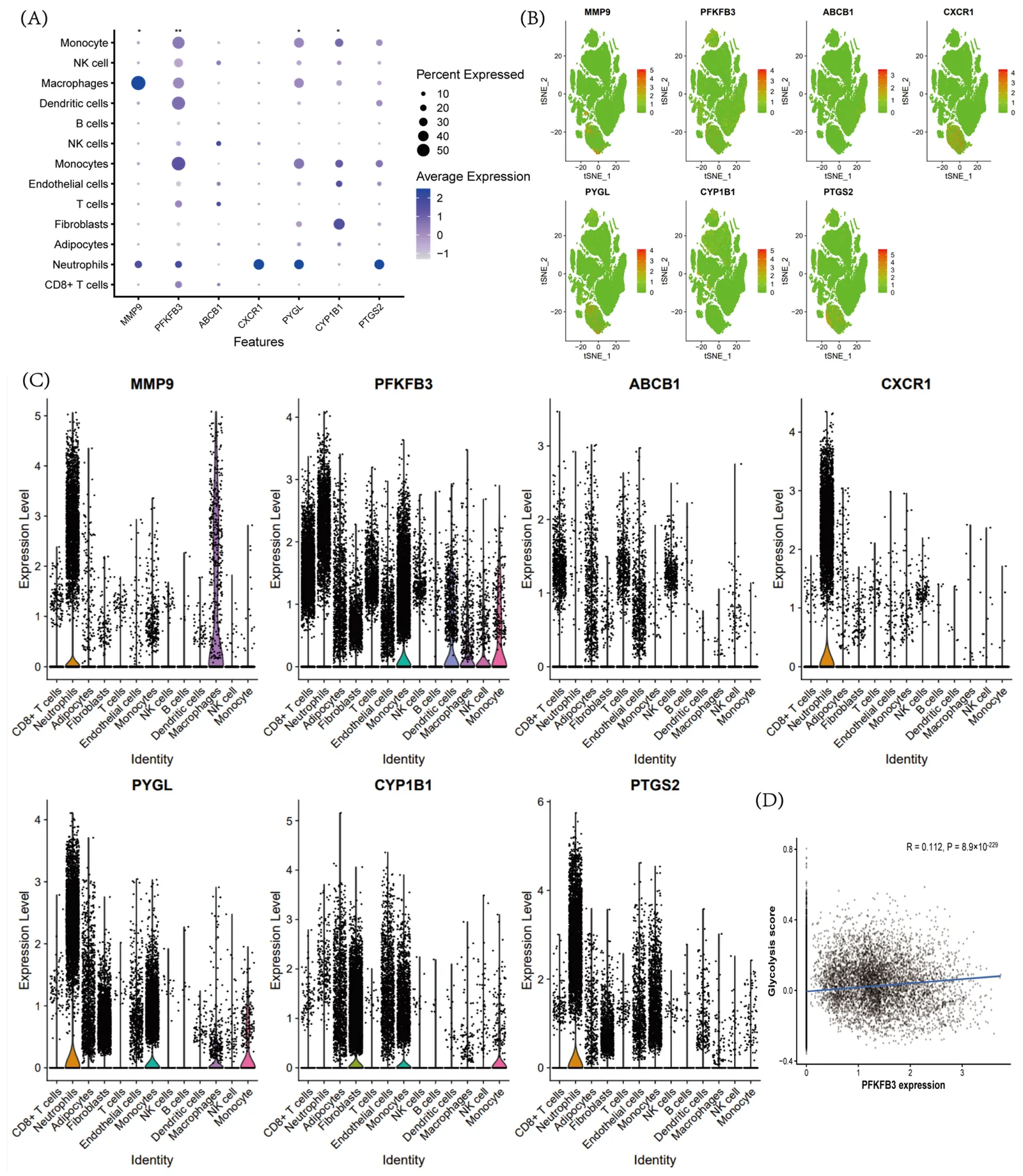
Cell-type-specific expression patterns of the seven diagnostic genes and prioritization of *PFKFB3* as a metabolically relevant hub gene. (**A**) Dot plot showing the expression distribution of the seven diagnostic genes across major PVAT cell populations. Dot size indicates the percentage of cells expressing each gene, and color intensity represents average expression level. (**B**) Feature plots showing the spatial expression patterns of the seven diagnostic genes across the t-SNE landscape. (**C**) Violin plots showing the expression levels of the seven diagnostic genes across distinct cell populations. (**D**) Correlation analysis between PFKFB3 expression and glycolysis score at the single-cell level. The glycolysis score was calculated after excluding PFKFB3 from the scoring gene set. PFKFB3 expression showed a modest but statistically significant positive association with glycolytic activity.

With the cellular landscape of PVAT characterized and the seven diagnostic genes identified, we next examined their cell-type-specific expression patterns at single-cell resolution in the GSE233870 dataset to evaluate their potential biological relevance. As shown in the dot plot, feature plots, and violin plots, the seven genes exhibited distinct cell-type-specific expression patterns rather than uniform distribution across all identified cell populations (Figure 4A–C). Specifically, MMP9, CXCR1, and PTGS2 showed relatively restricted expression in selected immune cell populations, whereas CYP1B1 and PYGL displayed higher expression in specific cellular subsets. In contrast, *PFKFB3* showed a relatively broader and more consistent expression pattern across multiple cell populations, with appreciable expression levels in several major immune and stromal cell subsets. These findings suggest that the diagnostic genes identified from the bulk transcriptomic data may exert their biological effects within distinct cellular contexts in CHD. To support the priority of PFKFB3 with quantitative evidence, seven diagnostic genes were systematically compared across four dimensions: feature selection frequency, differential expression amplitude, individual discrimination performance, and single-cell expression width (Supplementary Table S4). In short, *PFKFB3* was retained (100%) in all 113 algorithm combinations, significantly upregulated in CHD (log_2_FC = 1.369), ranked first in seven gene expressions, and demonstrated the widest single-cell expression distribution in the PVAT cell population with a validation AUC of 0.824. These quantitative indicators, combined with the maturation role of PFKFB3 as a glycolytic regulator, provide aggregated computational support for its priority as a central gene for downstream computer perturbation and functional enrichment analysis. Consistent with this role, *PFKFB3* expression showed a modest but statistically significant positive correlation with glycolysis activity at the single-cell level (Figure 4D). Among these candidates, *PFKFB3* was prioritized as the key regulatory hub gene for further analysis. This prioritization was based on three considerations. First, PFKFB3 was retained as one of the seven diagnostic features in the optimal machine learning model. Second, single-cell analysis showed that PFKFB3 was not confined to a highly restricted cellular compartment, but instead displayed a relatively broad distribution across multiple PVAT cell populations, supporting its potential role in intercellular regulatory processes. Third, given its reported involvement in metabolic reprogramming and inflammatory regulation, PFKFB3 was considered a biologically plausible hub gene for downstream analysis. In addition, PFKFB3 also showed differential expression in the bulk transcriptomic analysis, further supporting its prioritization for downstream investigation. It should be noted that this prioritization is based on converging computational evidence rather than formal causal testing; systematic in silico or experimental comparison of all seven genes would be required to conclusively establish PFKFB3 as the most central regulatory node.

Taken together, these findings suggest that PFKFB3 may serve as a biologically plausible hub gene linking diagnostic value with cell-context-dependent regulatory mechanisms in CHD. Therefore, PFKFB3 was selected for subsequent in silico perturbation analysis and downstream functional enrichment analysis. This prioritization was further supported by subsequent analyses linking PFKFB3 expression to glycolytic activity and inflammatory transcriptional programs.

### 2.5. Metabolic Pathway Activity Analysis Reveals a Metabolic–Transcriptional Link Associated with PFKFB3

**Figure 5:**
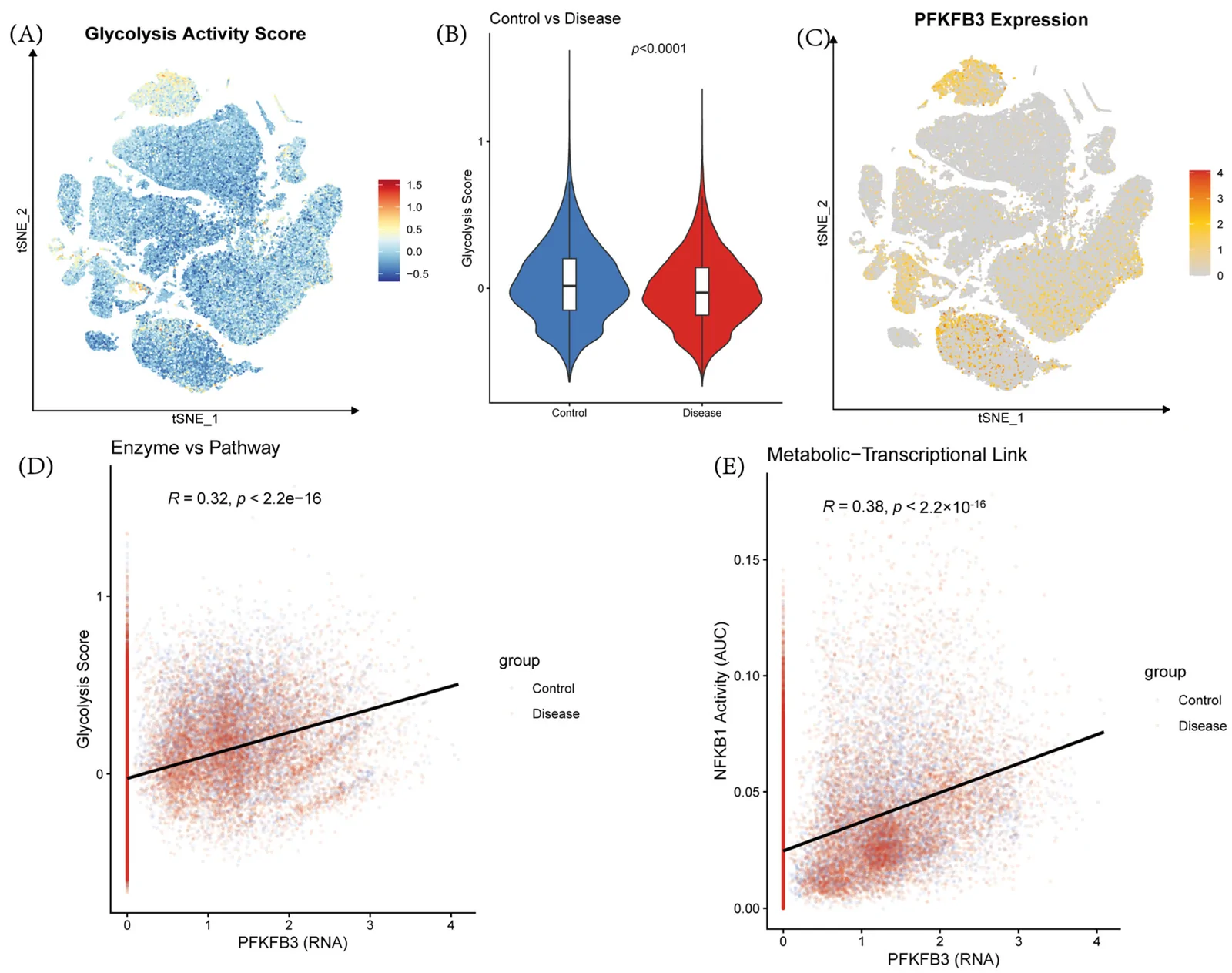
Metabolic pathway activity analysis of PFKFB3-associated glycolytic remodeling in CHD. (**A**) t-SNE visualization of glycolysis activity scores across single cells in PVAT, showing heterogeneous distribution of glycolytic activity across cell populations. (**B**) Violin plot comparing glycolysis activity scores between control and disease groups, highlighting significantly higher glycolytic activity in disease-associated cell populations (*p* < 0.0001). (**C**) t-SNE plot showing PFKFB3 expression across single cells, with enriched expression in macrophages and endothelial cells. (**D**) Correlation between *PFKFB3* RNA expression and glycolysis activity score, showing a significant positive correlation (R = 0.32, *p* < 2.2 × 10^−16^). (**E**) Correlation between PFKFB3 RNA expression and NF-κB transcriptional activity as measured by AUC scores, demonstrating a positive association (R = 0.38, *p* < 2.2 × 10^−16^), suggesting a metabolic–transcriptional link in CHD.

Given that PFKFB3 is a well-established glycolytic regulator, we next investigated whether its expression was functionally coupled to glycolytic pathway activity in the CHD microenvironment. We first computed glycolysis activity scores for each cell using a hallmark glycolysis gene set. t-SNE visualization revealed significant heterogeneity in glycolytic activity across distinct cell populations in PVAT, with higher glycolytic enzyme activity in macrophages and endothelial cells and lower activity in populations such as fibroblasts and adipocytes (Figure 5A).

Comparison between the Control and Disease groups showed that glycolytic activity was significantly increased in disease-associated cell populations. Specifically, macrophages and endothelial cells in the Disease group exhibited higher glycolysis activity compared with the Control group (*p* < 2.2 × 10^−16^), whereas fibroblasts and adipocytes showed no significant differences (Figure 5B). This suggests that glycolytic reprogramming in CHD is preferentially enriched in immune and endothelial cell populations, rather than occurring uniformly across all cell types.

PFKFB3, a key glycolytic enzyme, was widely expressed across various cell populations, with higher expression levels in macrophages and endothelial cells. Its expression pattern largely coincided with regions of high glycolytic activity (Figure 5C). Further correlation analysis revealed a significant positive correlation between *PFKFB3* expression and glycolysis activity (R = 0.32, *p* < 2.2 × 10^−16^; Figure 5D), suggesting that PFKFB3 expression is associated with enhanced glycolytic activity in CHD. We also investigated the potential transcriptional regulation of glycolytic activity by NF-κB, a transcription factor implicated in inflammation and metabolic processes. A positive correlation was observed between PFKFB3 expression and NFKB1 activity (AUC) (R = 0.38, *p* < 2.2 × 10^−16^; Figure 5E), indicating that PFKFB3-mediated metabolic alterations may be linked to inflammatory transcriptional programs in the CHD microenvironment. Although these correlations reached high statistical significance owing to the large number of single cells analyzed, their modest magnitudes (R = 0.32–0.38) indicate that PFKFB3 expression explains only a fraction of the variance in glycolysis and NF-κB activity, and should be interpreted as suggestive rather than definitive evidence of functional coupling.

Together, these results (Figure 5) indicate that PFKFB3 expression is closely associated with glycolytic activity in CHD, suggesting its potential involvement in glycolytic remodeling. Moreover, the observed correlation between *PFKFB3* expression and NF-κB activity suggests a potential link between metabolic changes and inflammation in disease-associated cell populations. Given the observed association—though modest in magnitude—between glycolytic activity and NF-κB signaling, we next investigated the transcriptional regulatory programs potentially underlying this metabolic shift.

### 2.6. Cell Type Specific Transcription Factor Regulatory Activity 

**Figure 6:**
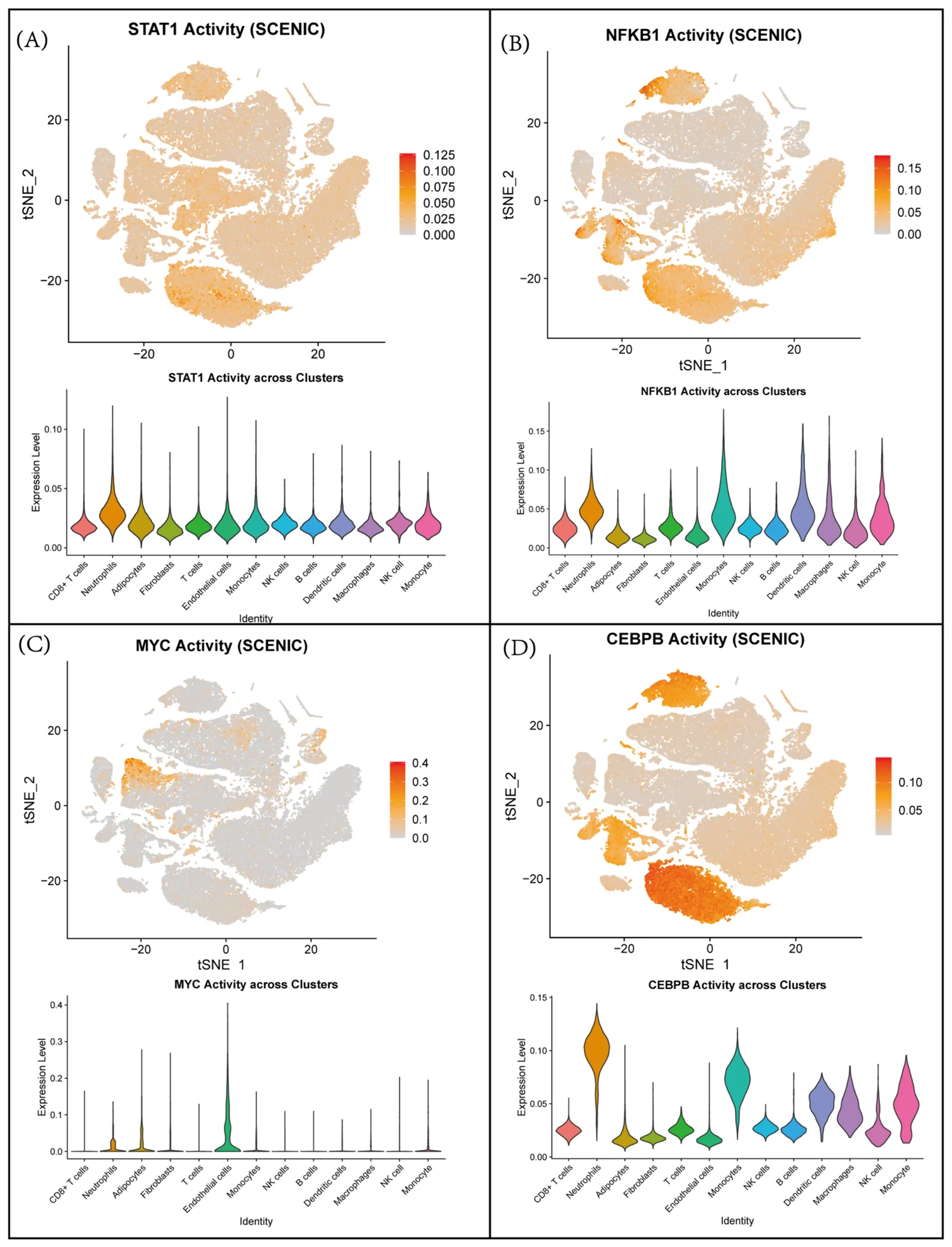
Transcription factor regulatory activity analysis based on SCENIC. (**A**) t-SNE visualization and violin plot showing STAT1 regulon activity across different cell populations. STAT1 activity was broadly distributed and relatively enriched in immune cell populations, suggesting activation of immune-related transcriptional programs. (**B**) t-SNE visualization and violin plot showing NF-κB regulon activity. NF-κB activity was increased in inflammatory immune populations, including neutrophils, monocytes, dendritic cells, and macrophages, indicating activation of NF-κB-related inflammatory regulation. (**C**) t-SNE visualization and violin plot showing MYC regulon activity. MYC activity was mainly enriched in endothelial cells, suggesting potential involvement in endothelial functional remodeling. (**D**) t-SNE visualization and violin plot showing CEBPB regulon activity. CEBPB activity was highly enriched in myeloid-lineage cells, including neutrophils, monocytes, macrophages, and dendritic cells, indicating its potential role in myeloid inflammatory activation.

The observed correlation between PFKFB3 expression and NF-κB activity suggested a potential link between metabolic remodeling and transcriptional regulation. To systematically characterize the transcriptional regulatory landscape across PVAT cell populations, we performed SCENIC-based regulon analysis. To characterize transcriptional regulatory changes across cell populations, SCENIC-based regulon activity was analyzed for key transcription factors. The results showed distinct cell-type-specific activation patterns of STAT1, NF-κB, MYC, and CEBPB, suggesting that different regulatory programs may participate in immune activation, inflammatory signaling, and cellular functional remodeling. STAT1 regulon activity was broadly distributed across the t-SNE map, with relatively higher activity in several immune-cell-enriched regions. Violin plot analysis showed that STAT1 activity was elevated in multiple cell populations, particularly in neutrophils, monocytes, dendritic cells, macrophages, and NK cell-related populations (Figure 6A). This suggests that STAT1-mediated transcriptional regulation may be involved in interferon-related immune activation within the disease microenvironment. NF-κB regulon activity displayed a more prominent activation pattern in inflammatory cell populations. The t-SNE distribution showed focal enrichment of NF-κB activity in several cell clusters, while the violin plot indicated higher activity in neutrophils, monocytes, dendritic cells, macrophages, and Monocyte populations (Figure 6B). These findings suggest that NF-κB-associated inflammatory transcriptional programs are activated in immune cells and may contribute to the pro-inflammatory state observed in CHD. MYC regulon activity showed a relatively restricted distribution compared with STAT1 and NF-κB. The t-SNE plot revealed localized MYC activation, and the violin plot showed that MYC activity was mainly enriched in endothelial cells, with only limited activity in other cell populations (Figure 6C). This indicates that MYC-related transcriptional regulation may be associated with endothelial cell functional changes, possibly reflecting altered proliferation, metabolism, or stress responses. CEBPB regulon activity showed strong enrichment in myeloid-lineage populations. Both the t-SNE map and violin plot demonstrated high CEBPB activity in neutrophils, monocytes, macrophages, dendritic cells, and related immune populations (Figure 6D). This pattern suggests that CEBPB may serve as an important regulator of myeloid cell activation and inflammatory remodeling in CHD.

Taken together, these results indicate that transcription factor regulatory activity is highly cell-type-specific. STAT1 and NF-κB were associated with broad immune inflammatory activation, MYC was mainly enriched in endothelial cells, and CEBPB showed strong activity in myeloid populations. These findings suggest that CHD-associated cellular remodeling is accompanied by coordinated activation of immune, inflammatory, endothelial, and myeloid transcriptional programs.

### 2.7. Cell–Cell Communication Analysis Revealed Enhanced Macrophage-Centered Signaling in CHD

**Figure 7:**
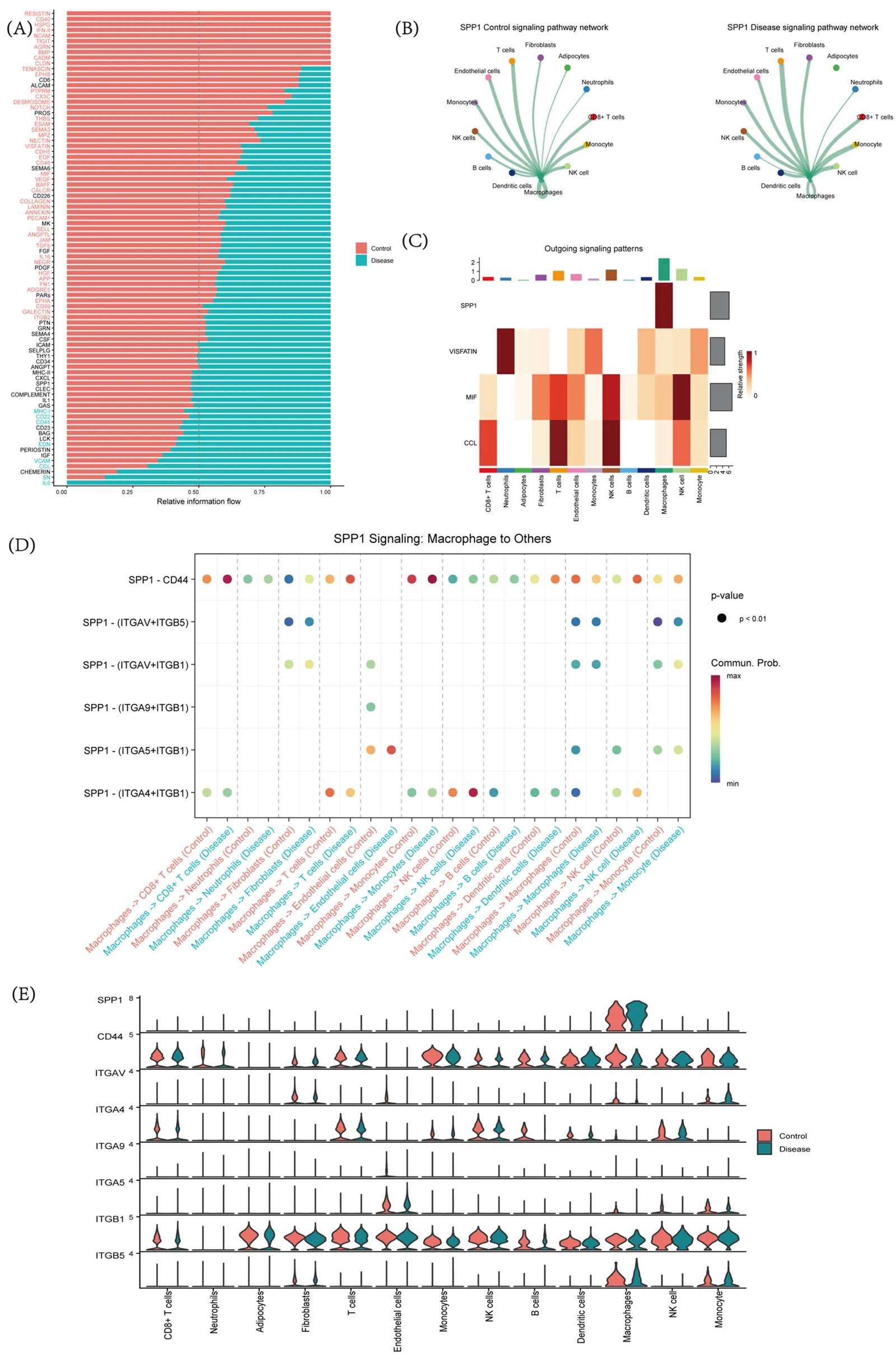
Cell–cell communication analysis of CHD-associated PVAT. (**A**) Relative information flow of signaling pathways between the Control and Disease groups, showing altered intercellular communication activity in CHD-associated PVAT. (**B**) Comparison of the SPP1 signaling pathway network between the Control and Disease groups. The Disease group showed a stronger macrophage-centered SPP1 communication pattern. (**C**) Outgoing signaling patterns of selected pathways, including SPP1, VISFATIN, MIF, and CCL, identifying macrophages as the major signal-sending population. (**D**) Bubble plot showing macrophage-derived ligand–receptor interactions across recipient cell populations. Major interactions included SPP1–CD44, SPP1–integrin complexes, MIF–CD74 receptor complexes, NAMPT-related interactions, and CCL–CCR1/ACKR1 interactions. (**E**) Expression distribution of SPP1 and its associated receptors, including CD44, ITGAV, ITGA4, ITGA9, ITGA5, ITGB1, and ITGB5, supporting the potential role of the SPP1–CD44/integrin axis in macrophage-mediated communication.

The cell-type-specific activation of immune and inflammatory transcriptional programs raised the question of whether these intracellular regulatory changes are accompanied by altered intercellular signaling. To further investigate whether CHD-associated metabolic and inflammatory remodeling was accompanied by alterations in intercellular communication, we performed CellChat analysis. The detailed visualization of intercellular communication networks, signaling pathway comparisons, and ligand–receptor interaction analyses is shown in Figure 7. The overall relative information flow showed that, compared with the Control group, the Disease group exhibited increased activity in multiple signaling pathways, particularly SPP1, MIF, VISFATIN, CCL, CXCL, and VEGF, indicating broad remodeling of inflammatory, chemotactic, and stromal communication programs in CHD-associated PVAT (Figure 7A). The SPP1 signaling pathway network showed that macrophages were the major source of SPP1-mediated communication in both groups; however, the Disease group displayed a denser and stronger macrophage-centered SPP1 network than the Control group (Figure 7B). This suggests that SPP1-mediated intercellular communication is enhanced in CHD and that macrophages may function as a key signaling hub in the disease microenvironment. Outgoing signaling pattern analysis further showed that macrophages had the strongest outgoing activity among the analyzed cell populations, especially in the SPP1 pathway, while VISFATIN, MIF, and CCL signaling also contributed to disease-associated communication remodeling (Figure 7C). This indicates that macrophages are the dominant signal-sending cells in the CHD communication network, particularly through pathways associated with inflammation and metabolic regulation. The ligand–receptor interaction bubble plot identified multiple macrophage-associated communication pairs, including CCL3–CCR1, CCL3L1–CCR1, CCL5–ACKR1, CCL5–CCR1, MIF–CD74/CD44, MIF–CD74/CXCR4, MIF–ACKR3, NAMPT–ITGA5/ITGB1, NAMPT–INSR, SPP1–ITGA4/ITGB1, SPP1–ITGA5/ITGB1, SPP1–ITGA9/ITGB1, SPP1–ITGAV/ITGB1, SPP1–ITGAV/ITGB5, and SPP1–CD44 (Figure 7D). These interactions mainly connected macrophages with CD8+ T cells, neutrophils, fibroblasts, T cells, endothelial cells, monocytes, NK cells, B cells, dendritic cells, and macrophages, suggesting that macrophages may broadly regulate immune and stromal compartments in CHD.

Finally, the expression distribution of SPP1 and its associated receptors showed that SPP1 was mainly centered on macrophage-derived signaling, whereas its receptors, including CD44, ITGAV, ITGA4, ITGA9, ITGA5, ITGB1, and ITGB5, were broadly expressed across multiple recipient cell populations (Figure 7E). This provides molecular support for widespread macrophage-to-multiple-cell communication through the SPP1–CD44/integrin axis in the Disease group.

### 2.8. In Silico Perturbation of PFKFB3 Reveals Downstream Transcriptional Changes

**Figure 8:**
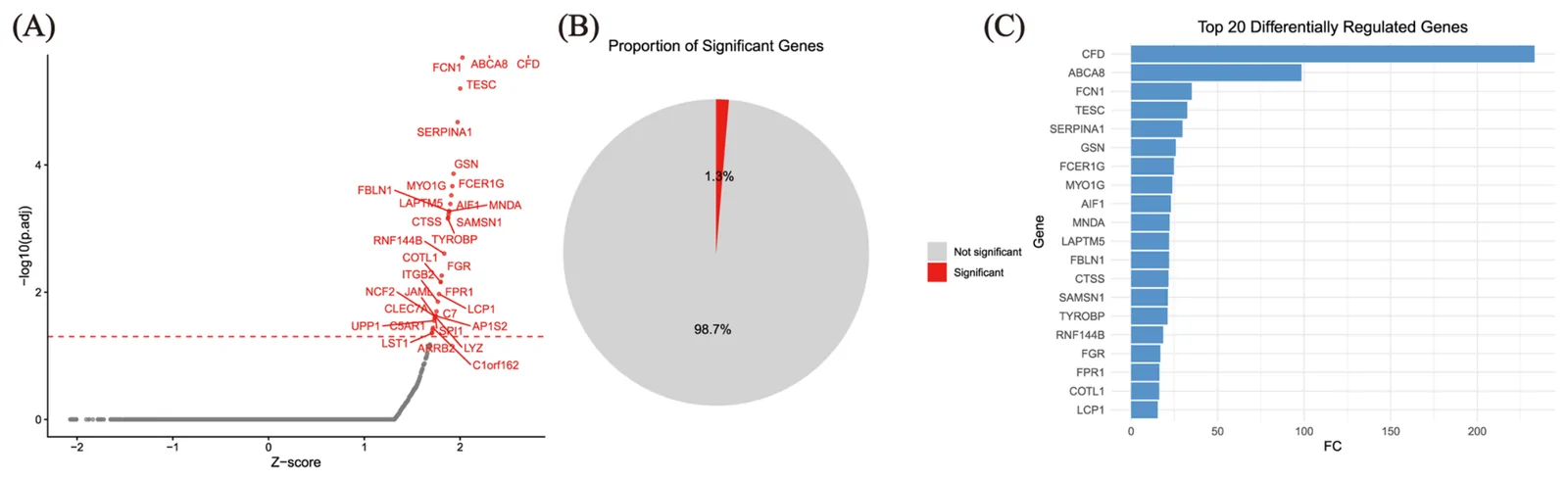
In silico perturbation analysis of PFKFB3 in the CHD microenvironment. (**A**) Proportion plot showing the percentage of significantly perturbed and non-significantly perturbed genes after virtual knockout of PFKFB3. The red dashed line indicates the adjusted *p* value threshold of 0.1 (−log10(0.1)). (**B**) Volcano plot showing the overall distribution of significantly perturbed genes following PFKFB3 virtual knockout. (**C**) Bar plot showing the top 20 significantly perturbed genes ranked by fold change after PFKFB3 perturbation.

The enhanced macrophage-centered communication and the central role of PFKFB3 in glycolytic–inflammatory coupling prompted us to investigate the downstream regulatory consequences of perturbing PFKFB3 in the CHD cellular microenvironment. To further investigate the regulatory role of PFKFB3 in the CHD microenvironment, a network perturbation-based in silico knockout analysis was performed to infer its potential downstream gene network. As shown in the proportion plot, only 1.3% of the analyzed genes exhibited significant perturbation after virtual knockout of PFKFB3 (adjusted *p* value < 0.1), whereas 98.7% of genes were not significantly affected (Figure 8A). This finding suggests that perturbation of PFKFB3 may induce a relatively focused transcriptional response rather than broad transcriptomic disruption. The volcano plot further illustrated the overall distribution of significantly perturbed genes following PFKFB3 knockout (Figure 8B). Both positively and negatively perturbed genes were observed, and several genes showed marked fold changes after virtual knockout. Consistent with the proportion analysis, only a small subset of genes reached statistical significance. While this may indicate that PFKFB3 regulates a relatively restricted downstream program, it should also be interpreted with caution: the limited perturbation breadth may reflect the inherent constraints of network-based in silico knockout, which relies on wild-type GRN topology and may underestimate indirect or compensatory regulatory effects. Accordingly, the 1.3% perturbation rate should be viewed as a lower-bound estimate of PFKFB3’s regulatory influence rather than a definitive measure of its biological impact.

To further identify the most relevant downstream targets associated with PFKFB3 perturbation, the top 20 significantly perturbed genes were selected according to fold change and visualized in a bar plot (Figure 8C). Among these genes, CFD, ABCA8, and FCN1 showed positive perturbation after virtual PFKFB3 knockout, suggesting that they may represent candidate downstream effectors associated with the transcriptional response to PFKFB3 loss. These top perturbed genes were subsequently used for downstream functional enrichment analysis. It should be emphasized that this analysis was conducted globally rather than in a cell-type-specific manner and that the results are hypothesis-generating: they identify candidate downstream pathways and processes that may be associated with PFKFB3 perturbation, but do not establish causal regulatory relationships, which require experimental validation.

### 2.9. Functional Enrichment Analysis of PFKFB3-Associated Perturbed Genes

**Figure 9:**
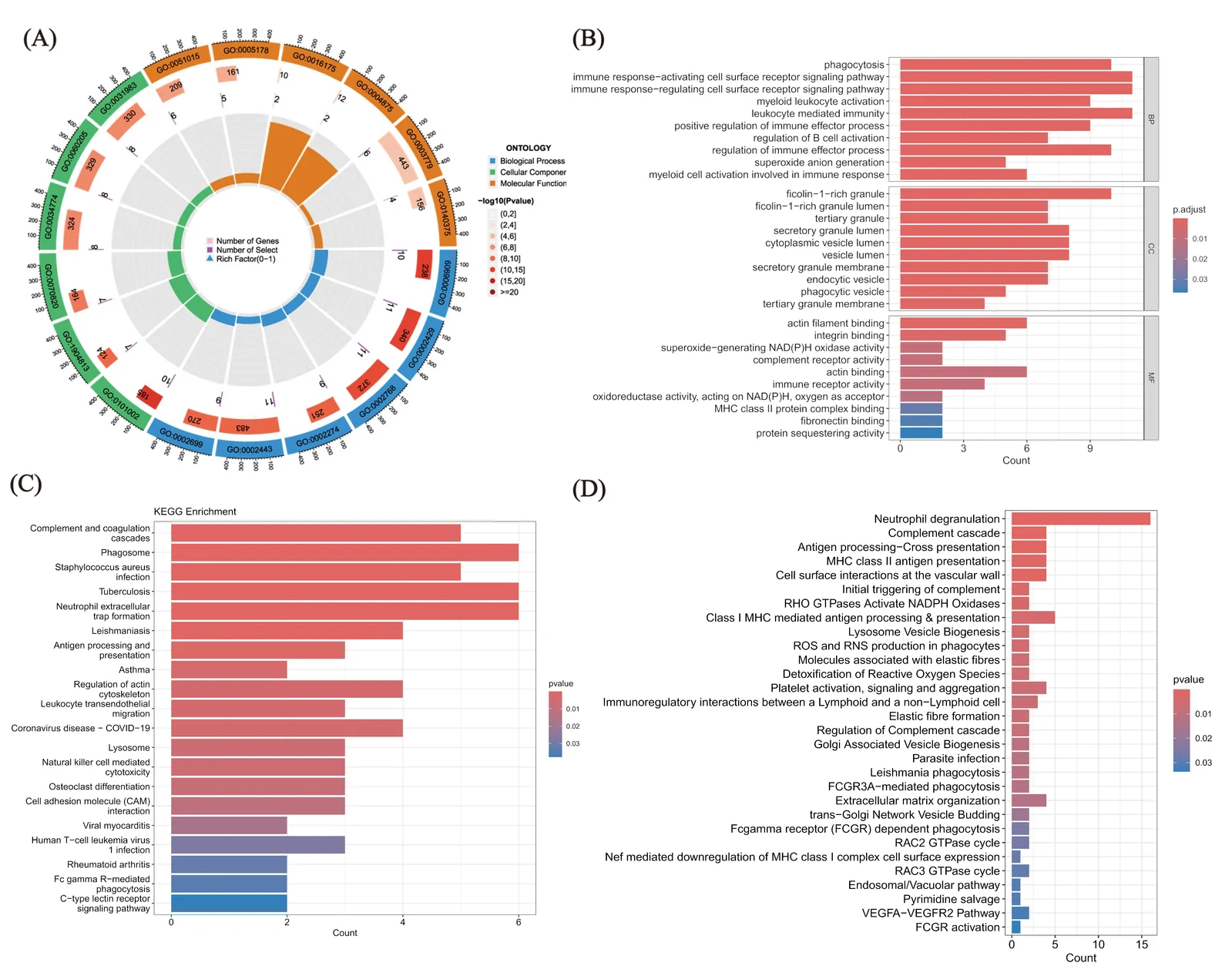
Functional enrichment analysis of PFKFB3-associated perturbed genes. (**A**) Overview of the enrichment analyses performed on the top 20 significantly perturbed genes identified after virtual knockout of PFKFB3. (**B**) Gene Ontology (GO) enrichment analysis showing the significantly enriched biological process, cellular component, and molecular function terms. (**C**) Kyoto Encyclopedia of Genes and Genomes (KEGG) pathway enrichment analysis of the significantly perturbed genes. (**D**) Reactome pathway enrichment analysis of the significantly perturbed genes.

To understand the biological significance of the transcriptional changes induced by PFKFB3 perturbation, we next performed functional enrichment analyses on the significantly perturbed genes. To further characterize the biological significance of the downstream transcriptional changes associated with PFKFB3 perturbation, functional enrichment analyses were performed on the top 20 significantly perturbed genes (ranked by |log_2_ fold change|) identified after virtual knockout. Gene Ontology (GO), Kyoto Encyclopedia of Genes and Genomes (KEGG), and Reactome analyses were conducted to explore the biological processes and pathways potentially associated with the PFKFB3-regulated downstream network in the CHD microenvironment (Figure 9A). GO enrichment analysis showed that these perturbed genes were mainly enriched in immune- and phagocytosis-related biological processes, including phagocytosis, immune response-activating cell surface receptor signaling pathway, immune response-regulating cell surface receptor signaling pathway, leukocyte mediated immunity, and myeloid leukocyte activation (Figure 9B). In the cellular component category, the enriched terms were primarily related to ficolin-1-rich granule, tertiary granule, secretory granule lumen, secretory vesicle lumen, and phagocytic vesicle, whereas the molecular function category was mainly enriched in MHC protein binding, superoxide-generating NADPH oxidase activity, complement receptor activity, and immune receptor activity. These findings suggest that PFKFB3 perturbation may influence immune effector functions, vesicle-associated structures, and oxidative response-related molecular activities. KEGG pathway analysis further revealed that the perturbed genes were significantly enriched in pathways associated with innate immunity and inflammatory regulation, including complement and coagulation cascades, phagosome, *Staphylococcus aureus* infection, neutrophil extracellular trap formation, antigen processing and presentation, and leukocyte transendothelial migration (Figure 9C). In addition, several immune-related or inflammatory disease-associated pathways, such as rheumatoid arthritis, Fc gamma R-mediated phagocytosis, and C-type lectin receptor signaling pathway, were also enriched.

Consistent with the GO and KEGG results, Reactome analysis showed that the perturbed genes were mainly involved in neutrophil degranulation, complement cascade, antigen processing cross-presentation, MHC class II antigen presentation, RHO GTPases activate NADPH oxidases, ROS and RNS production in phagocytes, and FCGR3A-mediated phagocytosis (Figure 9D). Collectively, these enrichment results indicate that the downstream transcriptional program associated with PFKFB3 perturbation is functionally linked to immune activation, phagocytic processes, antigen presentation, and oxidative stress-related responses in CHD. These findings provide additional support for the potential role of PFKFB3 as a biologically relevant hub gene in the CHD-associated microenvironment.

### 2.10. Molecular Dynamics Analysis of the Salidroside–PFKFB3 Complex

**Figure 10:**
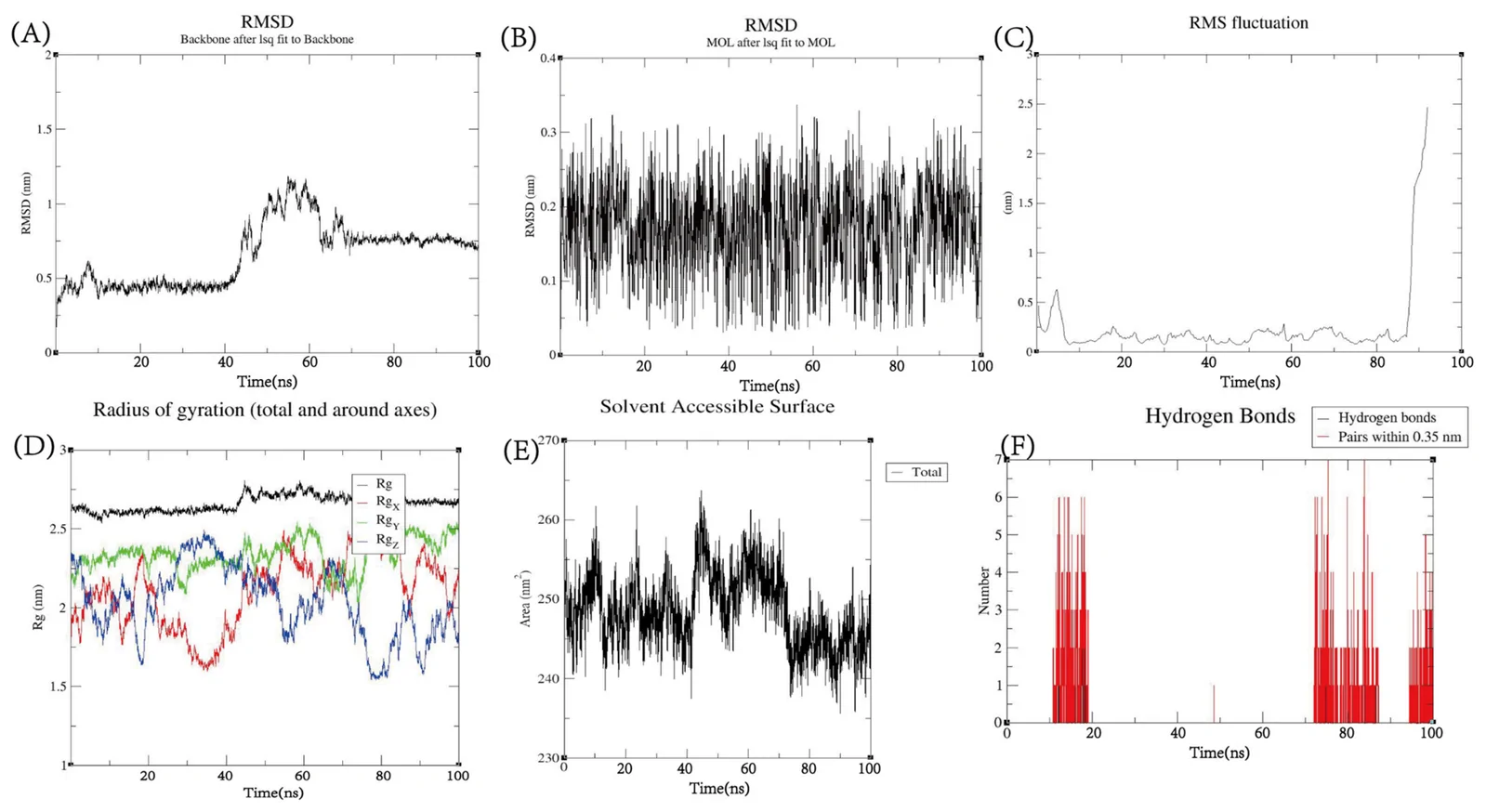
Salidroside–PFKFB3 molecular dynamics simulation. (**A**) RMSD of the PFKFB3 protein backbone over 100 ns, showing stabilization after ~50 ns. (**B**) RMSD of salidroside relative to PFKFB3, indicating confinement within the binding pocket. (**C**) Residue-wise RMSF, highlighting low fluctuations around the binding site and higher mobility in loop regions and termini. (**D**) Rg of the complex, demonstrating maintenance of protein compactness. (**E**) SASA over the simulation, confirming structural integrity. (**F**) Hydrogen bond analysis between salidroside and PFKFB3, showing persistent interactions throughout the trajectory.

The functional enrichment results indicated that PFKFB3 perturbation primarily affects immune effector and inflammatory pathways. Given that salidroside has been reported to modulate these same pathways and that previous network pharmacology analyses have suggested PFKFB3 as a potential salidroside target, we next sought to evaluate whether salidroside could structurally interact with PFKFB3 at the atomic level. To further evaluate the structural stability and binding behavior of salidroside with PFKFB3, molecular dynamics (MD) simulations were performed for 100 ns. The functional enrichment results indicated that PFKFB3 perturbation primarily affects immune effector and inflammatory pathways. Given that salidroside has been reported to modulate these same pathways and that previous network pharmacology analyses have suggested PFKFB3 as a potential salidroside target, we next sought to evaluate whether salidroside could structurally interact with PFKFB3 at the atomic level. To this end, molecular dynamics (MD) simulations were performed for 100 ns. Multiple complementary metrics were analyzed to assess the conformational dynamics and interaction persistence of the complex. Protein backbone RMSD analysis (Figure 10A) showed that the complex reached equilibrium after an initial equilibration phase, with relatively stable fluctuations throughout the remaining simulation, indicating maintenance of overall structural integrity. Ligand RMSD analysis (Figure 10B) revealed moderate fluctuations during the early stage of the simulation, reflecting conformational adaptation of salidroside within the binding pocket, followed by stabilization, suggesting the establishment of a confined binding mode. The radius of gyration (Rg) remained largely constant during the simulation (Figure 10D), demonstrating that the protein maintained a compact conformation without significant unfolding. Similarly, solvent-accessible surface area (SASA) analysis (Figure 10E) exhibited only minor fluctuations, further supporting the structural stability of the complex. Residue-level RMSF analysis (Figure 10C) indicated expected flexibility in loop regions and terminal segments, whereas residues surrounding the binding pocket displayed relatively low fluctuations, suggesting the formation of a stable interaction interface. Hydrogen bond analysis (Figure 10F) demonstrated persistent interactions between salidroside and key residues of PFKFB3 throughout the simulation, indicating sustained ligand binding.

Collectively, these results indicate that the salidroside–PFKFB3 complex exhibits stable dynamic behavior, supporting the structural plausibility of a potential interaction. These findings provide a structural rationale for the potential interaction between salidroside and PFKFB3, warranting further experimental investigation to determine whether salidroside modulates PFKFB3-associated glycolytic and inflammatory pathways in CHD.

### 2.11. Integrative Model of Glycolytic Reprogramming in CHD-Associated PVAT

**Figure 11:**
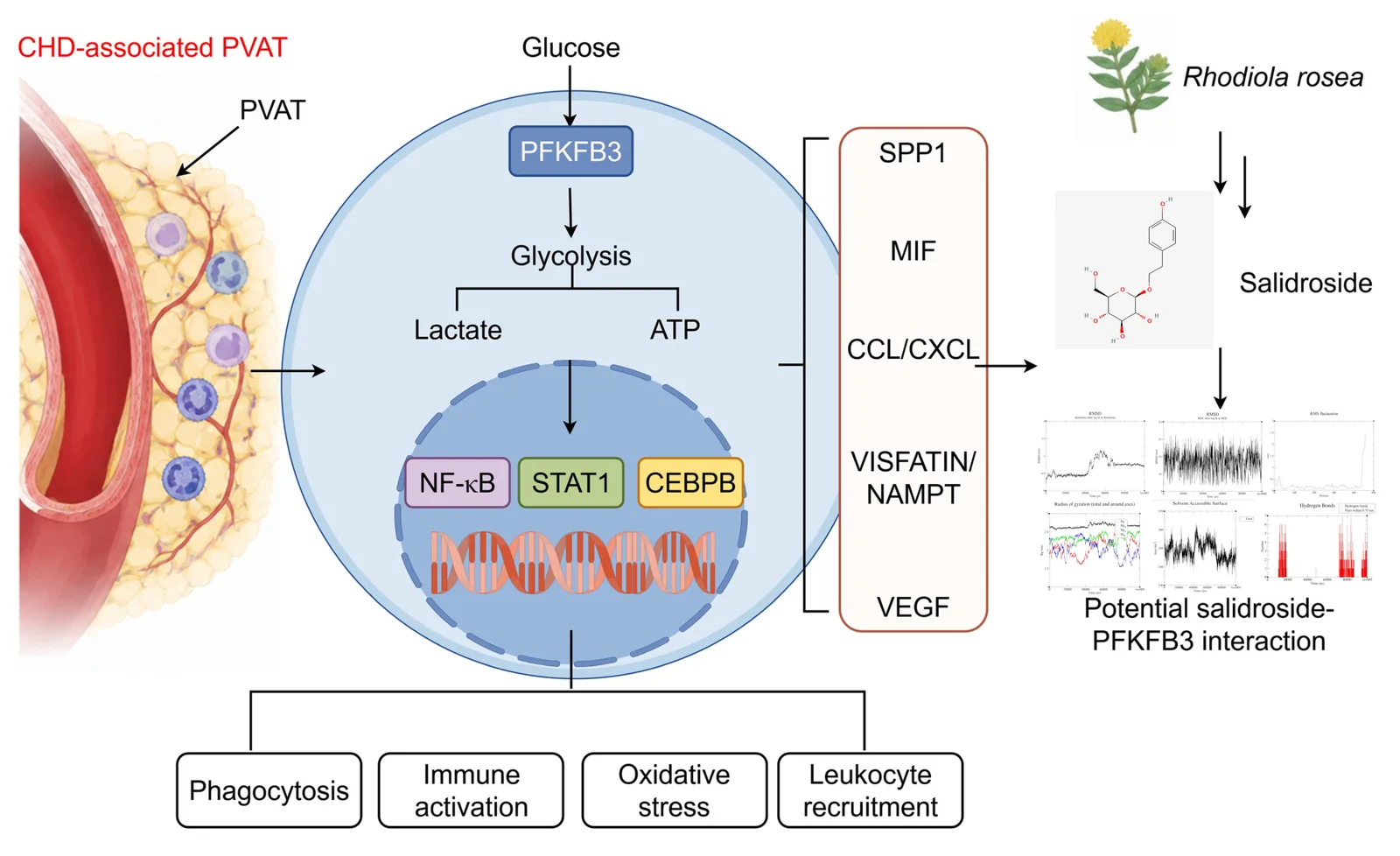
Schematic illustration of CHD-associated PVAT metabolic reprogramming via PFKFB3-driven glycolysis and the potential modulatory interaction by salidroside.

To integrate the multi-layered evidence from the preceding analyses into a coherent working model, we constructed a schematic illustration of PFKFB3-driven glycolytic reprogramming in CHD-associated PVAT. As shown in Figure 11, elevated glucose uptake in PVAT under CHD conditions leads to transcriptional and post-translational upregulation of PFKFB3. This rate-limiting enzyme increases the production of fructose-2,6-bisphosphate, thereby activating PFK1 and accelerating glycolytic flux. The resulting lactate accumulation acts as both a metabolic intermediate and a signaling molecule, promoting the activation of key transcription factors including NF-κB, STAT1, and CEBPB. Downstream, this cascade is predicted to promote pro-inflammatory phenotypes characterized by enhanced phagocytosis, immune cell activation, oxidative stress, and leukocyte recruitment to the perivascular space. Importantly, molecular dynamics simulations suggested stable interactions between salidroside and PFKFB3, supporting its potential to modulate the enzyme’s activity. This schematic model integrates transcriptomic and functional inferences from the present study; however, because intermediate processes including lactate accumulation, transcription factor activation, and inflammatory remodeling were inferred from computational analyses rather than directly measured, the model should be interpreted as a hypothesis-generating framework rather than an established mechanism. As shown in Figure 11, elevated glucose uptake in PVAT under CHD conditions leads to transcriptional and post-translational upregulation of PFKFB3. This rate-limiting enzyme increases the production of fructose-2,6-bisphosphate, thereby activating PFK1 and accelerating glycolytic flux. The resulting lactate accumulation acts as both a metabolic intermediate and a signaling molecule, promoting the activation of key transcription factors including NF-κB, STAT1, and CEBPB. Downstream, this cascade is predicted to promote pro-inflammatory phenotypes characterized by enhanced phagocytosis, immune cell activation, oxidative stress, and leukocyte recruitment to the perivascular space.

## 3. Discussion

The present study integrated bulk transcriptomics, machine learning, single-cell transcriptomics, glycolytic activity scoring, transcription factor regulon inference, cell–cell communication analysis, in silico perturbation, molecular dynamics simulation, and mechanistic visualization to characterize the transcriptomic and regulatory features of CHD-associated PVAT. The principal finding is that *PFKFB3* was not only retained as one component of a seven-gene diagnostic signature but also emerged as a biologically interpretable hub connecting glycolytic reprogramming, inflammatory transcriptional activation, macrophage-centered intercellular communication, and a potential salidroside-mediated intervention. A secondary but important finding is that CHD-associated remodeling was not uniformly distributed across all PVAT cell types; instead, metabolic and inflammatory signals were preferentially enriched in macrophages, endothelial cells, and myeloid-lineage compartments. This pattern suggests that PVAT dysfunction in CHD may be driven by cell-type-specific metabolic–immune remodeling rather than by a nonspecific tissue-wide transcriptional disturbance. Given the continuing global burden of CHD and the increasing recognition that vascular inflammation, endothelial dysfunction, and metabolic remodeling jointly contribute to disease progression, these findings provide a mechanistic extension beyond conventional risk-factor-centered interpretations of CHD [1,8,12,13,14].

The biological plausibility of these findings is supported by the central role of PFKFB3 in controlling glycolytic flux. PFKFB3 promotes the generation of fructose-2,6-bisphosphate, a potent allosteric activator of PFK1, thereby enhancing glycolytic throughput and supporting rapid energy production and biosynthetic adaptation under inflammatory or hypoxic stress [15,16]. In the vascular microenvironment, glycolysis is not merely an energy-producing process; it actively participates in shaping endothelial activation, monocyte/macrophage recruitment, inflammatory cytokine production, and vascular remodeling [17].

The positive, albeit modest, associations among *PFKFB3* expression, glycolysis activity (R = 0.32), and NF-κB regulon activity (R = 0.38) suggest—but do not confirm—that PFKFB3 may participate in a metabolic–transcriptional feed-forward circuit in CHD-associated PVAT. In such a circuit, inflammatory stimulation may increase glycolytic demand; PFKFB3-dependent glycolysis may provide metabolic support for immune activation; and glycolytic products or downstream metabolic signals may further reinforce inflammatory transcriptional programs. Recent evidence has linked endothelial metabolic remodeling and lactate-related signaling to vascular inflammation and atherosclerotic progression, supporting the possibility that glycolysis-derived metabolites can function as signaling mediators rather than passive by-products [18]. In this context, the integrated model shown in Figure 11 provides a working hypothesis for the present results: increased glucose uptake and PFKFB3 activation may enhance PFK1-dependent glycolysis, promote lactate accumulation, and contribute to the activation of NF-κB, STAT1, and CEBPB-associated inflammatory programs. However, because lactate accumulation was inferred from glycolytic pathway activity rather than directly measured by metabolomics, this part of the model should be interpreted as a plausible mechanistic hypothesis rather than definitive biochemical evidence.

The cell–cell communication results extend this interpretation from intracellular metabolism to intercellular regulation. The increased SPP1, MIF, VISFATIN, CCL, CXCL, and VEGF signaling in the disease group suggests that CHD-associated PVAT may act as an active immunometabolic niche. In particular, macrophages appeared to be the dominant signal-sending population, especially through SPP1-related interactions. This finding is consistent with recent single-cell evidence showing that SPP1-positive macrophage subpopulations in coronary PVAT may contribute to local inflammatory and fibrotic remodeling [19]. SPP1–CD44 and SPP1–integrin interactions may promote macrophage retention, extracellular matrix remodeling, stromal activation, and endothelial–immune crosstalk, whereas MIF–CD74/CXCR4/ACKR3 signaling may reinforce immune recruitment, inflammatory survival signaling, and vascular inflammation [20]. The enrichment of PFKFB3 perturbation-associated genes in phagocytosis, antigen processing and presentation, complement activation, neutrophil degranulation, and ROS/RNS production further supports the idea that PFKFB3 is embedded in immune effector programs rather than being limited to canonical glycolysis.

The present findings are broadly consistent with previous experimental and single-cell studies. PFKFB3 has been reported to be increased in vulnerable human atherosclerotic plaques and to be mainly localized in macrophages and endothelial cells; pharmacological inhibition of PFKFB3 reduced plaque progression and promoted plaque stability in experimental atherosclerosis [21]. Studies of macrophage metabolism have shown that HIF-1α and PFKFB3 can link pro-inflammatory activation with anaerobic metabolism in atherosclerotic macrophages, while *PFKFB3* gene dosage influences monocyte/macrophage biology in atherosclerosis [22]. Recent work further indicates that hypoxia can activate macrophage NLRP3 inflammasome signaling through PFKFB3-driven glycolysis, supporting the connection among hypoxia, glycolysis, inflammasome activation, and atherosclerotic inflammation [23]. The present study agrees with these observations by showing that PFKFB3 is associated with glycolysis activity and inflammatory transcriptional activation at single-cell resolution. Our results are also consistent with evidence that endothelial PFKFB3 contributes to vascular inflammatory phenotypes and endothelial dysfunction in cardiometabolic disease models [24]. Meanwhile, recent reports showing that natural compounds such as resveratrol, luteolin, and ginsenoside Re can modulate endothelial injury or atherosclerosis-related glycolytic pathways provide external support for the broader therapeutic relevance of targeting metabolic inflammation in vascular disease [25].

At the same time, the literature also suggests that the role of PFKFB3 is not simply linear or universally harmful. Some studies indicate that PFKFB3 inhibition may stabilize advanced plaque features, whereas others suggest that endothelial PFKFB3 deletion can aggravate vascular inflammation or produce context-dependent effects [26]. Similarly, recent evidence that glucose metabolism controls monocyte homeostasis and migration but does not necessarily reduce atherosclerosis development after systemic modulation highlights the compensatory complexity of immune–metabolic networks [27]. These apparently divergent findings share an important common point: PFKFB3 function depends on cell type, disease stage, dosage, local metabolic reserve, and mode of perturbation. This context-dependence helps explain why the present study identifies PFKFB3 as a regulatory hub and hypothesis-generating target, rather than presenting it as a simple monotherapeutic target. It also implies that future therapeutic strategies should avoid indiscriminate glycolytic suppression and instead consider moderate, cell-type-specific, and stage-aware modulation of PFKFB3-centered pathways.

The rationale for progressing from transcriptomic and single-cell analyses to molecular dynamics simulation merits explicit discussion. The multi-omics analyses established *PFKFB3* as a regulatory hub at the intersection of glycolysis, inflammation, and intercellular communication in CHD-associated PVAT. However, these correlative findings, while biologically coherent, do not address whether salidroside can physically engage PFKFB3. Molecular dynamics simulation was therefore employed not as an isolated experiment but as a complementary structural approach to test whether the salidroside–PFKFB3 interaction is physically plausible at the atomic level. This progression from correlation to structural plausibility represents a deliberate analytical strategy: transcriptomic evidence generates the hypothesis, single-cell analysis provides cellular context, and molecular dynamics offers an initial structural filter before committing to resource-intensive experimental validation. The salidroside-related findings provide another layer of mechanistic and translational relevance. Salidroside has been reported to exert anti-inflammatory, antioxidant, endothelial-protective, and cardioprotective effects in multiple experimental contexts [28]. Of particular relevance to the present study, salidroside has demonstrated direct antiatherosclerotic effects in experimental models through attenuation of lipid accumulation, reduction in inflammatory cytokine production, and improvement of endothelial function [29,30]. Mechanistically, salidroside-mediated activation of Nrf2/HO-1 signaling has been identified as a key pathway through which herbal medicine protects vascular endothelial cells from oxidative stress in atherosclerosis [31]. Furthermore, emerging evidence suggests that salidroside may modulate gut microbiota composition as an additional mechanism contributing to its antiatherosclerotic effects and has been implicated in the regulation of pyroptosis—an inflammatory form of programmed cell death increasingly recognized in atherosclerotic plaque progression [32]. In the present study, molecular dynamics simulation suggested that salidroside could remain stably positioned within the PFKFB3 binding pocket, providing structural plausibility for a potential interaction between salidroside and PFKFB3. However, no direct comparison was made between salidroside and known PFKFB3 inhibitors (e.g., 3PO, PFK15) regarding binding mode, affinity, or target engagement [33,34]. Such comparative analyses would be necessary to assess whether salidroside represents a plausible pharmacological modulator of PFKFB3 relative to established inhibitors. Combined with the transcriptomic and single-cell results, this supports the hypothesis that salidroside may interfere with the PFKFB3-centered glycolytic–inflammatory loop summarized in Figure 11. Nevertheless, this conclusion should be interpreted cautiously. Molecular dynamics simulation can support binding stability and generate a mechanistic hypothesis, but it cannot prove direct binding affinity, enzymatic inhibition, intracellular target engagement, or in vivo therapeutic efficacy. Therefore, the salidroside–PFKFB3 axis proposed here should be considered a promising but experimentally unconfirmed direction.

The theoretical significance of this study lies in its integration of diagnostic modeling with cell-contextual mechanism inference. Unlike studies that identify predictive signatures without biological interpretation, this multi-layered strategy transforms PFKFB3 from a model-selected feature into a biologically interpretable hub, linking diagnostic prediction to mechanistic hypothesis generation. Methodologically, this framework is consistent with recent trends in causal and integrative single-cell genomics, which emphasize moving beyond marker discovery toward regulatory interpretation and perturbation-based analysis [35].

From a practical perspective, the seven-gene diagnostic signature may provide a candidate tool for CHD risk stratification after validation in independent and prospective cohorts. If future studies confirm that PFKFB3-high macrophages or endothelial cells mark metabolically activated inflammatory niches in PVAT, PFKFB3 could be used for patient stratification, therapeutic monitoring, or drug response prediction. The proposed salidroside–PFKFB3 interaction may also provide a starting point for investigating natural-product-based modulation of glycolytic inflammation. In addition, recent advances in targeted delivery systems for atherosclerosis suggest that future therapeutic strategies may combine metabolic targets with cell- or lesion-directed drug delivery, thereby improving efficacy while reducing systemic metabolic adverse effects [36,37,38].

Several limitations should be acknowledged. First, this study was based on public retrospective datasets, and the bulk transcriptomic cohorts and single-cell PVAT dataset were not derived from the same individuals. This design increases data availability and enables cross-platform analysis, but it may introduce heterogeneity related to population background, disease stage, sample source, platform differences, and clinical treatment status. As a result, the findings should be interpreted as convergent computational evidence rather than direct patient-level causal proof. Although ComBat correction effectively reduced the principal batch effects between GSE66360 and GSE179789 (Figure 1C,D), residual confounding from unmeasured variables—including differences in patient demographics, disease severity, medication regimens, and sample processing protocols—cannot be fully excluded. Furthermore, the seven-gene diagnostic signature was validated in a single independent dataset (GSE24519, n = 34). Although the validation AUC (0.846) was acceptable, additional validation in larger, prospective cohorts with standardized clinical covariates, medication histories, and cardiovascular outcome data is necessary before the signature can be considered for clinical application. Future multi-center cohorts with matched transcriptomic, single-cell, spatial, and clinical outcome data are needed to confirm the robustness of the diagnostic signature and PFKFB3-centered mechanism. Second, the single-cell analysis was limited by the cellular coverage and donor-level sample size of the available PVAT dataset (n = 10 donors; 4 controls, 6 CHD). Cell-level statistical comparisons do not fully account for within-donor correlation, and the reported p values should be interpreted as exploratory. Future studies with larger donor cohorts should employ pseudobulk aggregation or mixed-effects models to confirm cell-type-specific expression differences at the donor level. Rare cell populations, transitional states, or disease-stage-specific subclusters may therefore have been underdetected, which could affect the resolution of cell-type-specific regulatory inference. Spatial transcriptomics and multiplex immunofluorescence would help determine whether PFKFB3-high macrophages, endothelial cells, and SPP1/MIF-enriched signaling niches are anatomically organized around diseased coronary vessels. Third, glycolysis activity, transcription factor activity, and cell–cell communication were inferred from transcriptomic data rather than directly measured functional assays. Although these computational methods are widely used, they cannot fully substitute for metabolic flux assays, protein-level validation (e.g., qPCR, Western blot, immunohistochemistry), or ligand–receptor perturbation experiments. Fourth, scTenifoldKnk-based virtual knockout provides a network-level prediction of PFKFB3 perturbation effects but does not replace experimental knockout or knockdown. Cell-type-specific PFKFB3 silencing, CRISPR interference, overexpression, and rescue experiments in macrophages and endothelial cells are needed to determine whether PFKFB3 causally regulates downstream phenotypes. Fifth, molecular dynamics simulation supports structural stability but does not establish binding affinity, target engagement, enzymatic regulation, or therapeutic efficacy; future validation should include surface plasmon resonance (SPR), isothermal titration calorimetry (ITC), cellular thermal shift assays (CETSA), PFKFB3 enzymatic assays, and in vivo pharmacokinetic/pharmacodynamic experiments. Sixth, although 113 algorithm combinations were compared using five repeats of ten-fold cross-validation, hyperparameter tuning was performed using commonly used parameter settings rather than exhaustive grid search, and a fully nested cross-validation framework (with an inner loop for model selection and an outer loop for performance estimation) was not implemented. Consequently, the reported AUC values may represent slightly optimistic estimates of model performance, and the relative ranking of algorithms should be prioritized over absolute AUC values when interpreting the results. Seventh, the prioritization of PFKFB3 among the seven diagnostic genes was based on integrative computational evidence—including its retention in the optimal model, broader single-cell expression distribution, and known metabolic regulatory functions—rather than on systematic pairwise comparison or experimental validation of each candidate gene’s functional centrality. The other six genes may also contribute to CHD pathogenesis through distinct mechanisms that were not explored in this study.

Future research should therefore proceed from computational convergence toward experimental and translational validation. First, the seven-gene diagnostic model should be tested in larger prospective CHD cohorts with standardized clinical covariates, medication information, and cardiovascular outcome follow-up. Second, spatial transcriptomics and multiplex imaging should be used to validate whether PFKFB3-high macrophages and endothelial cells are spatially coupled to SPP1/MIF-enriched inflammatory regions in coronary PVAT and atherosclerotic plaques. Third, integrated metabolomics, proteomics, phosphoproteomics, and isotope-tracing experiments should determine whether PFKFB3 expression corresponds to actual glycolytic flux, lactate generation, and inflammatory transcriptional activation. Fourth, cell-type-specific perturbation experiments should clarify whether PFKFB3 regulates macrophage phagocytosis, antigen presentation, ROS production, and endothelial inflammatory activation in a causal manner. Fifth, salidroside should be evaluated using target engagement, dose–response, and rescue experiments to determine whether its protective effects depend on PFKFB3-mediated glycolytic remodeling. Taken together, this study provides a sustainable research framework for moving from computational biomarker discovery toward mechanism-guided therapeutic exploration in CHD-associated PVAT.

## 4. Materials and Methods

### 4.1. Data Sources and Preprocessing

Bulk transcriptomic datasets related to coronary heart disease (CHD), including GSE66360, GSE179789, and GSE24519, were obtained from the Gene Expression Omnibus (GEO) database (https://www.ncbi.nlm.nih.gov/geo/ (accessed on 11 August 2026)) [10,11]. GSE66360 and GSE179789 were used as the training cohort, while GSE24519 served as an independent validation cohort. Raw expression data from the training datasets were preprocessed by background correction, log2 transformation, and normalization using the normalizeBetweenArrays function in the limma R package. The normalized matrices were subsequently merged, and batch effects were corrected using the ComBat algorithm [39] implemented in the sva R package (v3.44.0), with dataset identity specified as the batch variable. ComBat was selected because its empirical Bayes framework is well-suited for batch correction with small-to-moderate sample sizes and has been widely validated in multi-cohort microarray integration studies [40]. Principal component analysis (PCA) was performed before and after batch correction to evaluate normalization effectiveness. The validation dataset GSE24519 was processed using the same normalization pipeline but was not included in batch correction or differential expression analysis. For cellular heterogeneity analysis, single-cell RNA sequencing (scRNA-seq) data (GSE233870) derived from PVAT of CHD patients and healthy controls were also downloaded. The gene–cell count matrix generated by Cell Ranger (v7.3.1) was used for downstream single-cell analyses [41].

### 4.2. Identification of Differentially Expressed Genes

Differential expression analysis was performed on the batch-corrected training dataset using the limma R package (v3.52.4) [42]. A linear model was constructed to compare gene expression profiles between CHD and control groups. Genes with a false discovery rate (FDR) < 0.05 and an absolute log_2_ fold change (|log_2_FC|) > 1 were considered significantly differentially expressed. Identified DEGs were visualized using volcano plots and hierarchical clustering heatmaps.

### 4.3. Machine Learning Based Feature Selection and Model Construction

To identify robust diagnostic features for CHD, machine learning analysis was performed using the DEGs identified in the training cohort as candidate input features. The training cohort (GSE66360 + GSE179789) comprised 123 samples (58 controls, 65 CHD), and the independent validation cohort (GSE24519) comprised 38 samples (4 controls, 34 CHD). Prior to model training, DEGs (n = 203; |log_2_FC| > 1, FDR < 0.05) were identified from the training cohort only; the validation cohort was held out entirely from differential expression analysis, feature selection, and model training to prevent information leakage. The expression levels of candidate genes were standardized using Z-score normalization and used as input features, while disease status served as the classification label. A total of 113 machine learning algorithms, comprising combinations of feature selection methods (LASSO, glmBoost, Random Forest, and Stepwise GLM) and classifiers (Gradient Boosting Machine, Random Forest, Support Vector Machine, Linear Discriminant Analysis, XGBoost, Naive Bayes, Ridge regression, LASSO, Elastic Net with α = 0.1–0.9, Stepwise GLM [forward, backward, and both directions], glmBoost, and partial least squares regression for GLM), were trained and compared. A total of 113 machine learning algorithms, each comprising a feature selection method paired with a classifier, were systematically trained and compared. Feature selection was performed de novo within each cross-validation fold to avoid optimistic bias: for each fold, feature ranking or regularization was applied exclusively to the training partition, and the selected features were then evaluated on the held-out partition. The complete list of 113 algorithm combinations—consisting of four feature selection methods (LASSO, glmBoost, Random Forest, and Stepwise GLM) each paired with one of thirteen classifiers (Gradient Boosting Machine, Random Forest, Support Vector Machine, Linear Discriminant Analysis, XGBoost, Naive Bayes, Ridge regression, LASSO, Elastic Net with α = 0.1–0.9, Stepwise GLM [forward, backward, and both directions], glmBoost, and partial least squares regression for GLM)—is provided in Supplementary Table S2. All algorithms were evaluated in the training cohort using ten-fold cross-validation with five repeats to ensure robust performance estimation. To reduce the influence of class imbalance, synthetic minority over-sampling was applied to the training partition of each cross-validation fold; the validation partition within each fold was never resampled. The area under the receiver operating characteristic curve (AUC) was used as the primary metric for model selection. Among all evaluated models, the Gradient Boosting Machine (GBM) achieved the best overall performance and was therefore selected as the optimal diagnostic model. The model parameters were set as follows: learning rate = 0.1, subsample rate = 0.8, maximum tree depth = 5, and number of trees = 100. These values were selected based on commonly used defaults for moderate-sample-size transcriptomic classification tasks. A formal exhaustive grid search or Bayesian optimization was not performed; this is acknowledged as a limitation. Based on model performance, feature importance ranking, and feature retention in the optimal model, seven genes—*PYGL*, *PTGS2*, *PFKFB3*, *MMP9*, *CYP1B1*, *CXCR1*, and *ABCB1*—were identified as the final diagnostic features and carried forward for downstream analyses. The optimized GBM model was then retrained on the entire training dataset and further evaluated in the independent validation cohort. To further evaluate model robustness, the training-versus-validation AUC gap was compared across all 113 algorithm combinations, and the convergence of the top five algorithms on an identical seven-gene signature was verified. Model performance was assessed by ROC analysis and AUC calculation. Decision curve analysis (DCA) was additionally performed to evaluate the clinical net benefit of the model, and a nomogram was constructed to facilitate individualized risk prediction. Furthermore, the diagnostic performance of each of the seven genes as an individual biomarker was evaluated in the validation cohort, and their expression differences between the CHD and control groups were assessed using the Wilcoxon rank-sum test.

### 4.4. Single-Cell Transcriptomic Analysis

The GSE233870 dataset, containing 10X Genomics scRNA-seq data derived from PVAT of CHD patients and healthy controls, was analyzed using the Seurat package (v4.5.0) [43]. Strict quality control procedures were applied, retaining cells with 200–6000 detected genes and a mitochondrial gene proportion < 20%. These thresholds were selected according to previously reported scRNA-seq quality control strategies and were used to remove low-quality or damaged cells. The data were subsequently normalized using the LogNormalize method, and 2000 highly variable genes were identified using the vst method. To remove batch effects among samples, the IntegrateData function based on canonical correlation analysis (CCA) was applied for dataset integration. The integrated dataset was then subjected to PCA for dimensionality reduction. A shared nearest neighbor (SNN) graph was constructed based on the top 20 principal components, and cell clustering was performed using the Louvain algorithm with a resolution parameter of 0.6. Cell clusters were annotated by comparing the expression of known cell type-specific marker genes. The annotation results were further automatically validated using the SingleR package (v1.8.1) [44] with the Human Primary Cell Atlas as the reference dataset. The final clustering results were visualized using t-distributed stochastic neighbor embedding (t-SNE) [45]. For the core targets, their expression distributions across different cell subpopulations were visualized using FeaturePlot and VlnPlot. Differences in target gene expression between the CHD and control groups within key cell types were assessed using the Wilcoxon rank-sum test, with FDR < 0.05 considered statistically significant. The resulting *p* values for each target within specific cell types were used to assess cell type-specific expression differences between CHD and control samples.

These cell-level comparisons should be interpreted as exploratory because cells from the same donor are not independent observations. The GSE233870 dataset comprises 10 donors (4 controls, 6 CHD). Donor identity was included as a batch variable during Seurat CCA integration to harmonize inter-donor technical variation. For key findings (e.g., PFKFB3 expression in macrophages and endothelial cells), results were confirmed at the pseudobulk level by aggregating expression to donor-level means per cell type and comparing groups. Cell-level p values are likely anti-conservative and are reported as descriptive rather than inferential.

### 4.5. Glycolytic Activity Analysis

To assess glycolytic activity at the single-cell level, glycolysis activity scores were calculated using a hallmark glycolysis gene set and the AddModuleScore function in Seurat. For correlation analyses between PFKFB3 expression and glycolysis activity, PFKFB3 was excluded from the scoring gene set to avoid circular inference. The resulting scores reflected the relative glycolytic activity of individual cells and were visualized on t-SNE embeddings to evaluate their distribution across PVAT cell populations. This function computes the activity of predefined gene sets at the single-cell level and assigns scores that reflect the relative glycolytic activity in each cell. The glycolysis scores were visualized using t-SNE to capture the global distribution of glycolytic activity across cells. Additionally, to examine the differences in glycolytic activity between the Control and Disease groups, a violin plot was generated to compare the glycolysis scores across these two conditions. The statistical significance of the differences between groups was evaluated using the Wilcoxon rank-sum test. To explore the potential role of PFKFB3, a key regulator of glycolysis, in the glycolytic activity of cells, the expression of PFKFB3 was visualized across the t-SNE map. The correlation between *PFKFB3* expression and glycolytic activity was assessed by performing a Pearson correlation analysis at the single-cell level. To assess potential transcriptional regulation, the activity of NF-κB, a transcription factor associated with inflammation and metabolic reprogramming, was also measured. The transcriptional activity of NF-κB was evaluated using AUC scores derived from the pySCENIC (v0.12.1), and the correlation between PFKFB3 expression and NF-κB activity was analyzed to explore a potential connection between metabolic and transcriptional changes in the CHD microenvironment.

### 4.6. Transcription Factor Regulatory Activity Analysis

Transcription factor regulatory activity was assessed using pySCENIC-derived AUCell scores and the corresponding Seurat object. The AUCell matrix was matched to the Seurat object based on shared cell barcodes and incorporated as a SCENIC assay. Regulon activities were visualized on UMAP and t-SNE embeddings using FeaturePlot, and cluster-level distributions were examined using VlnPlot. Cluster-specific active regulons were identified using FindAllMarkers with only.pos = TRUE, min.pct = 0.1, and logfc.threshold = 0.01. The top five regulons per cluster were selected according to average log2 fold change. Regulon activity patterns were further visualized using DoHeatmap after ScaleData, and average regulon activity across clusters was summarized using AverageExpression and displayed with pheatmap (v1.0.12).

### 4.7. Cell–Cell Communication Analysis

Intercellular signaling in CHD was assessed using CellChat (v1.6.1) on the merged Seurat object containing Control and Disease groups, with RNA expression as the default assay and cell types as group labels. To reduce computational load while preserving representation, each cell type was downsampled to a maximum of 500 cells. For each group, a CellChat object was created and assigned the human CellChat database. Overexpressed genes and ligand–receptor interactions were identified using identifyOverExpressedGenes and identifyOverExpressedInteractions, and communication probabilities were computed via computeCommunProb with the triMean method, retaining interactions with at least 10 cells per population. Probabilities were aggregated at the signaling pathway level using compute Commun Prob Pathway and aggregate Net. Control and Disease objects were merged with merge CellChat for comparative analyses. Network centrality was computed using net Analysis compute Centrality to identify key signal senders and receivers. A set of candidate inflammatory, chemotactic, metabolic, and angiogenic pathways, including SPP1, VISFATIN, MIF, CCL, CXCL, and VEGF, was selected for focused downstream analysis. All computations were performed using an 8-worker parallelized framework, and resulting objects were saved for subsequent visualization and statistical evaluation.

### 4.8. Criteria for Identification of Key Regulatory Hub Genes

To identify key regulatory hub genes, the seven diagnostic features obtained from the machine learning model were further evaluated by integrating four criteria: (1) feature selection frequency across all 113 algorithm combinations, (2) differential expression statistics in the training cohort (|log_2_FC| and FDR), (3) individual discriminatory performance in the independent validation cohort, and (4) single-cell expression breadth across PVAT cell populations (see Supplementary Table S4). Genes that satisfied these criteria and showed potential biological relevance to CHD pathogenesis were prioritized as candidate hub genes. Among these candidates, genes with preferential expression in disease-relevant cell populations and known associations with metabolic or inflammatory regulation were considered for downstream in silico perturbation analysis.

### 4.9. In Silico Perturbation Analysis of the Selected Hub Gene

To investigate the potential regulatory role of the selected hub gene, in silico perturbation analysis was performed using the scTenifoldKnk algorithm (v1.0.1) [38]. Briefly, scTenifoldKnk constructs a single-cell gene regulatory network (GRN) from the wild-type expression matrix using principal component regression. PFKFB3 is then virtually deleted by removing its corresponding row and column from the GRN adjacency matrix, producing a pseudo-knockout GRN. Manifold alignment is subsequently performed between the wild-type and pseudo-knockout GRNs to quantify the transcriptional displacement of each gene. Genes whose network positions are significantly shifted after virtual knockout (FDR < 0.1) are identified as differentially perturbed and ranked by fold change. This approach enables gene function inference without requiring experimental knockout data, and has been validated against results from real-animal knockout experiments. The inferred knockout state was compared with the corresponding control state to quantify the transcriptional response. Differentially perturbed genes were identified at FDR < 0.1. This relaxed threshold was chosen because scTenifoldKnk produces conservative perturbation estimates from GRN topology alone and may underestimate indirect or compensatory effects; FDR < 0.1 was also used in the original scTenifoldKnk validation study. The analysis was performed globally (all cells combined) rather than per cell type, as scTenifoldKnk requires a single expression matrix and per-cluster network construction is unreliable for rare populations. The top 20 perturbed genes, ranked by absolute fold change, were selected for downstream functional enrichment analysis. This threshold was chosen because these genes exhibited the most pronounced transcriptional responses to PFKFB3 perturbation (FDR < 0.1) and are therefore most likely to reflect biologically meaningful downstream effects. In pathway enrichment analysis, including a large number of weakly perturbed or noise-level genes can dilute true enrichment signals and reduce statistical power; focusing on the top-ranked genes by effect size is a standard practice in perturbation-based functional genomics studies [38].

### 4.10. Functional Enrichment Analysis

To systematically elucidate the biological functions associated with the downstream transcriptional perturbation of PFKFB3, Gene Ontology (GO), Kyoto Encyclopedia of Genes and Genomes (KEGG), and Reactome pathway enrichment analyses were performed on the top 20 perturbed genes (ranked by absolute fold change; see Section 4.9 for selection rationale) identified after virtual knockout using the clusterProfiler R package v4.10.0 [46]. Enrichment analyses were conducted using the human genome as the background, and statistical significance was evaluated based on the default enrichment testing framework implemented in clusterProfiler. Multiple testing correction was performed using the Benjamini–Hochberg method, and enriched terms were considered significant when adjusted *p* value < 0.05 and enrichment fold > 2. The enrichment results were subsequently visualized using the ggplot2 package v3.5.2.

### 4.11. Molecular Dynamics Simulation

To further assess the binding stability of salidroside with PFKFB3, molecular dynamics (MD) simulations were performed using GROMACS 2022.3 [47] following previously established molecular dynamics simulation protocols. The crystal structure of PFKFB3 was retrieved from the Protein Data Bank using PDB ID 5AJV. The initial three-dimensional conformation of salidroside was obtained from the PubChem database and preprocessed with AmberTools 22 [48]. Atom types of salidroside were assigned using the General Amber Force Field (GAFF), hydrogen atoms were added, and restrained electrostatic potential (RESP) charges were calculated using Gaussian 16W; the resulting charges were incorporated into the ligand topology file. The PFKFB3 protein structure was prepared and modeled with the CHARMM36 force field [49]. The salidroside–PFKFB3 complex was placed in a cubic simulation box with the complex centered and a minimum distance of 1.0 nm between the solute and the box boundary. The system was solvated with water molecules and subsequently neutralized by adding Na^+^ and Cl^−^ ions as required to achieve overall charge neutrality. Energy minimization was performed using the steepest descent algorithm to remove steric clashes and unfavorable atomic contacts. Position restraints were generated for the ligand with a force constant of 1000 kJ mol^−1^ nm^−2^ in the x, y, and z directions. An index file was generated to define protein, ligand, and protein–ligand complex groups for equilibration and subsequent trajectory analyses. After energy minimization, the system was equilibrated under the canonical ensemble (NVT) at 300 K to stabilize the temperature, followed by isothermal–isobaric ensemble (NPT) equilibration at 1 bar to stabilize system pressure and density. The equilibrated system was then subjected to a 100 ns production MD simulation. Periodic boundary condition artifacts were removed by trajectory post-processing. The trajectory was first converted to maintain whole molecules, and the protein was subsequently centered to generate a continuous and properly aligned trajectory for downstream analyses.

Structural stability and interaction persistence of the salidroside–PFKFB3 complex were evaluated using multiple trajectory-based metrics. Protein backbone root-mean-square deviation (RMSD) was calculated after fitting to the backbone atoms to assess global protein stability. Ligand RMSD was calculated after protein alignment to evaluate the positional stability of salidroside within the binding pocket. Residue-level root-mean-square fluctuation (RMSF) was calculated to assess local flexibility of PFKFB3 residues. The radius of gyration (Rg) was computed to evaluate protein compactness, and solvent-accessible surface area (SASA) was calculated to monitor changes in solvent exposure. Hydrogen bond analysis was performed between PFKFB3 and salidroside to quantify protein–ligand hydrogen bond formation throughout the simulation.

## 5. Conclusions

In conclusion, this study identifies a seven-gene diagnostic signature for CHD and prioritizes PFKFB3 as a metabolically relevant hub linking glycolytic remodeling, inflammatory transcriptional activity, macrophage-centered communication, immune effector responses, and a potential salidroside interaction. By integrating bulk and single-cell transcriptomics with machine learning, regulatory inference, intercellular communication analysis, virtual perturbation, and molecular dynamics simulation, the study provides a multi-layered computational framework for understanding the metabolic–inflammatory microenvironment of CHD-associated PVAT. These findings support PFKFB3 as a candidate biomarker and hypothesis-generating target, while emphasizing that prospective cohort validation and experimental confirmation of PFKFB3 function and salidroside target engagement are required before clinical translation.

## Data Availability

All datasets analyzed in this study are publicly available from the Gene Expression Omnibus (GEO) database (https://www.ncbi.nlm.nih.gov/geo/ (accessed on 11 August 2026)) under accession numbers GSE66360, GSE179789, GSE24519, and GSE233870. The complete list of 203 differentially expressed genes, 113 machine learning algorithm combinations, and full performance metrics are provided in Supplementary Tables S1–S3.

## Supplementary Materials

The following supporting information can be downloaded at: https://www.mdpi.com/article/10.3390/ijms27167413/s1.

## Abbreviations

CHD	Coronary heart disease
PFKFB3	6-phosphofructo-2-kinase/fructose-2,6-bisphosphatase 3
scRNA-seq	single-cell RNA sequencing
PVAT	perivascular adipose tissue
DEGs	Differentially Expressed Genes
GEO	Gene Expression Omnibus
PCA	Principal Component Analysis
FDR	false discovery rate
GBM	Gradient Boosting Machine
AUC	Area Under the Curve
ROC	Receiver Operating Characteristic
DCA	Decision Curve Analysis
GO	Gene Ontology
KEGG	Kyoto Encyclopedia of Genes and Genomes
ABCB1	ATP Binding Cassette Subfamily B Member 1
AUCell	Automated Cell scoring
CCA	Canonical Correlation Analysis
CCL	C-C Motif Chemokine Ligand
CD	Cluster of Differentiation
CEBPB	CCAAT/Enhancer Binding Protein Beta
CXCL	C-X-C Motif Chemokine Ligand
CXCR1	C-X-C Motif Chemokine Receptor 1
CYPs	Cytochrome P450 Family
CYP1B1	Cytochrome P450 Family 1 Subfamily B Member 1
FC/Fold Change	Fold Change
GAFF	General Amber Force Field
Log2FC	Log2 Fold Change
MD	Molecular Dynamics
MIF	Macrophage Migration Inhibitory Factor
MMP9	Matrix Metallopeptidase 9
MYC	MYC Proto-Oncogene
NPT	Isothermal–Isobaric Ensemble
NVT	Canonical Ensemble
PTGS2	Prostaglandin-Endoperoxide Synthase 2
PYGL	Liver Glycogen Phosphorylase
RESP	Restrained Electrostatic Potential
Rg	Radius of Gyration
RMSD	Root-Mean-Square Deviation
RMSF	Root-Mean-Square Fluctuation
ROS/RNS	Reactive Oxygen Species/Reactive Nitrogen Species
SASA	Solvent-Accessible Surface Area
SCENIC	Single-Cell rEgulatory Network Inference and Clustering
SNN	Shared Nearest Neighbor
SPP1	Secreted Phosphoprotein 1
t-SNE	t-Distributed Stochastic Neighbor Embedding
UMAP	Uniform Manifold Approximation and Projection
VEGF	Vascular Endothelial Growth Factor
VISFATIN	Visfatin
